# New oxadiazole and pyrazoline derivatives as anti-proliferative agents targeting EGFR-TK: design, synthesis, biological evaluation and molecular docking study

**DOI:** 10.1038/s41598-024-55046-0

**Published:** 2024-03-05

**Authors:** Marwa I. Serag, Samar S. Tawfik, Sahar M. I. Badr, Hassan M. Eisa

**Affiliations:** Department of Pharmaceutical Organic Chemistry, Faculty of Pharmacy, Mansoura University, Mansoura, 35516 Egypt

**Keywords:** Oxadiazole, Pyrazoline, Anticancer activity, EGFR kinase, Docking studies, Cancer, Chemistry

## Abstract

Two new series of oxadiazole and pyrazoline derivatives were designed and synthesized as promising EGFR-TK inhibitors. The in vitro antiproliferative activity was studied against three human cancer cell lines; HCT116, HepG-2 and MCF7 using MTT assay. Compound **10c** showed the most potent anticancer activity against all cancer cell lines, with IC_50_ range of 1.82 to 5.55 μM, while proving safe towards normal cells WI-38 (IC_50_ = 41.17 μM) compared to the reference drug doxorubicin (IC_50_ = 6.72 μM). The most active candidates **5a, 9b, 10a, 10b** and **10c** were further assessed for their EGFR-TK inhibition. The best of which, compounds **5a** and **10b** showed IC_50_ of 0.09 and 0.16 μM respectively compared to gefitinib (IC_50_ = 0.04 μM). Further investigation against other EGFR family members, showed that **5a** displayed good activities against HER3 and HER4 with IC_50_ values 0.18 and 0.37 µM, respectively compared to gefitinib (IC_50_ = 0.35 and 0.58 µM, respectively). Furthermore, **5a** was evaluated for cell cycle distribution and apoptotic induction on HepG-2 cells. It induced mitochondrial apoptotic pathway and increased accumulation of ROS. Molecular docking study came in agreement with the biological results. Compounds **5a** and **10b** showed promising drug-likeness with good physicochemical properties.

## Introduction

The amendment in normal body cell proliferation may result in cancer development and its abruptly moving forward^1^. It is regarded as a terrible health issue that is responsible for high percent of mortality on a global scale^2^. Tyrosine kinases control several cellular processes like migration, angiogenesis, differentiation and proliferation, their over expression leads to cancer^3^. EGFR is a member of the ErbB family of receptor tyrosine kinases, also known as the epidermal growth factor (EGF) receptor family or type I receptor family which consists of four members: ErbB‐1/EGFR, ErbB‐2/ HER‐2/neu, ErbB‐3/HER‐3, and ErbB‐4/HER‐4^4^. Specific ligands including epidermal growth factor and transforming growth factor α (TGFα) attach to the extracellular domain of EGFR to cause dimerization, autophosphorylation and activation of the cytoplasmic tyrosine kinase domains^5^. Numerous human cancers, including breast, liver, colon, and prostate proved to have EGFR overexpression^6^. This overexpression correlates with vascularity and is linked to a poor prognosis^7^. Cancer therapy utilizing EGFR inhibitors has fewer negative effects since it primarily and exclusively kills cancer cells. That is why they are useful target for the development of a significant class of prospective anticancer drugs^8^.

**Figure 1:**
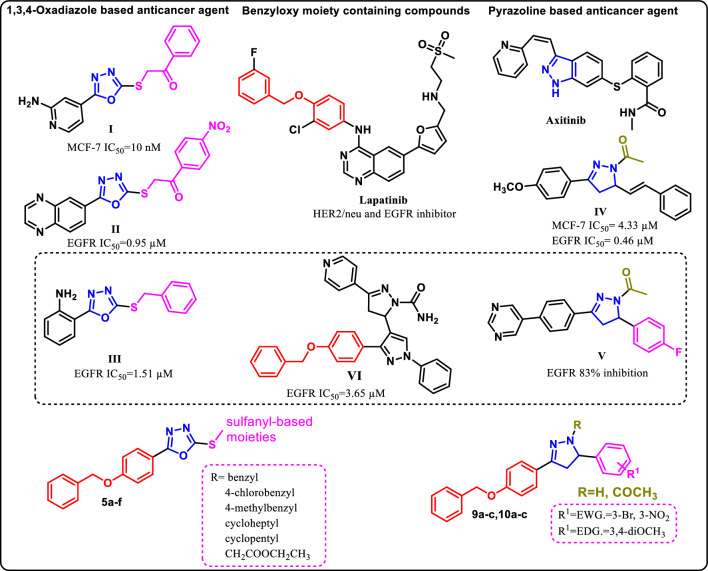
A 1,3,4-oxadiazole, pyrazoline, benzyloxy moiety containing compounds with reported anticancer activity and the structure of the target compounds **5a-f, 9a-c** and **10a-c**.

The 1,3,4-oxadiazole moiety has long been an important scaffold in drug design and synthesis with versatile biological activities, including hypoglycemic^9^, anti-fungal^10^, and anti-inflammatory activities ^11^. Several 1,3,4-oxadiazole derivatives have been reported for their cytotoxic action through numerous pathways and fortunately none of them has been linked to any negative side effects^12^. Particularly 2,5-disubstituted 1,3,4-oxadiazoles have become important strategy to create novel heterocyclic compounds, exhibiting a broad range of anticancer activities^13^. From literature survey, many compounds based on 2,5-disubstituted 1,3,4-oxadiazole scaffold have been synthesized and their anticancer properties were investigated^14^. For example, the activity investigations of compounds **I-III** showed potent activity levels between 10 nM and 1.51 μM^14–16^ (Fig. 1). The structure activity relationship (SAR) observations about 2,5-disubstituted 1,3,4- oxadiazole derivatives and their anticancer activities showed an aryl bulky group on the second position and an aromatic ring linked by an aliphatic chain on the fifth position are important for pharmacological activity in these compounds^14–16^. Moreover, according to reports, sulfanyl-based moiety exhibits significant efficacy as EGFR inhibitors against a number of cancer cell lines^14^.

Furthermore, the pyrazoline is a prevalent structural motif included in many pharmaceutically active compounds^17^. Numerous compounds based on 4,5-dihydropyrazole have demonstrated a variety of biological functions, such as anti-tumor^18^, anti-inflammatory^19^, and antimicrobial^20^, Many of them are currently being tested and/or clinically evaluated for new drug discovery. Axitinib is a pyrazoline-marketed second-generation tyrosine kinase inhibitor that functions by specifically blocking the vascular endothelial growth factor receptor (VEGFR)^21^. In addition, many series of new compounds containing the pyrazoline scaffold with an acetyl group attached to the nitrogen atom, called *N-acetyl* pyrazolines were reported to produce excellent cytotoxic activity targeting EGFR tyrosine kinase^22^. For example, compound **IV** displayed potent cytotoxic activity against breast cancer cell line (MCF-7) with IC_50_ of 4.33 µM, whereas, the positive control staurosporine showed IC_50_ of 10.61 µM, also exhibited submicromolar inhibitory activity of EGFR (IC_50_ = 0.46 μM) comparable to erlotinib (IC_50_ = 0.23 μM)^23^. Additionally, compound **V** has been managed to inhibit the EGFR enzyme with 83% and 82% of the inhibition rate for 0.19 and 50 μM concentrations, respectively ^24^.

Moreover, the molecular docking analysis of the reported EGFR inhibitor **VI** revealed that the presence of the bulky benzyloxy moiety helped in increasing EGFR kinase inhibitory activity as it introduced in a way identical to the orientation of ethynylphenyl of erlotinib and the oxygen atom of benzyloxy group involved in hydrogen bonding with the active site^25^. Notably, benzyloxy phenyl moiety is included in an orally active lapatinib, a dual tyrosine kinase inhibitor that inhibits the EGFR and HER2/neu pathways^26^.

Inspired by these data and via application of hybridization and bioisosteric modification strategies, this work focuses on the synthesis of new 2,5-disubstituted 1,3,4-oxadiazoles derivatives **5a-f** with (benzyloxy)phenyl) substituent as a bulky aromatic group in the second position and miscellaneous sulfanyl-based moieties differ in being unsubstituted aromatic (benzyl), aromatic with electron withdrawing group EWG (4-chlorobenzyl), aromatic with electron donating group EDG (4-methylbenzyl), cycloalkane (cycloheptyl and cyclopentyl) and finally linear aliphatic (CH_2_COOCH_2_CH_3_) in an attempt to obtain new potent antitumor agents with good bioavailability and low toxicity. Also, we aim to synthesis new 4,5-dihydro-1*H*-pyrazol-1-yl)ethan-1-one **9a-c** and 4,5-dihydro-1*H*-pyrazole **10a-c** derivatives with (benzyloxy)phenyl incorporated at position 2. Furthermore, different substituents varying in being EWG (Br, NO_2_) or EDG (OCH_3_) in the phenyl ring at fifth position were used to investigate the impact of their electronic effects on the obtained activity.

All compounds were synthesized in good yields and characterized by spectral data including IR, ^1^HNMR, ^13^CNMR, elemental analysis and mass spectrometry. They were then evaluated for their anti-proliferative activity against carefully selected three cell lines namely: colorectal carcinoma HCT-116, hepatocellular carcinoma HepG-2 and breast MCF-7 cancer cell lines in which EGFR is expressed^27^. All synthesized compounds were tested for their safety using WI-38. EGFR inhibition assay was performed. The most potent inhibitor **5a** was tested to study its effect on cell cycle progression and its ability to induce apoptosis on liver cancer cell line. Modeling studies have been applied to understand the obtained results on a molecular level. ADMET analysis for the most active compounds was also determined to further assess their drug like properties.

## Results and discussion

### Chemistry

**Figure 2:**
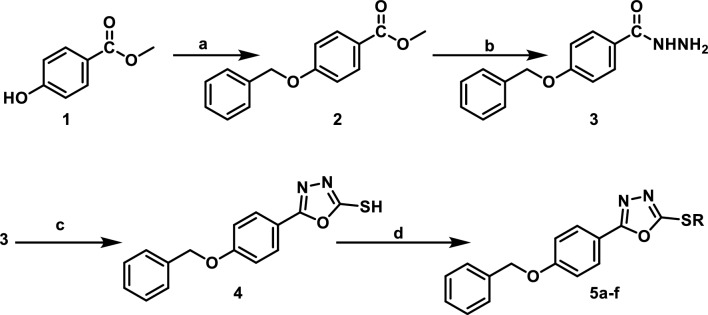
Synthesis of the target 1,3,4-oxadiazole derivatives **5a-f**. Reagents and Conditions: (**a**) benzyl chloride, K_2_CO_3_/DMF, r.t.; (**b**) hydrazine hydrate/abs. Ethanol, reflux; (**c)** KOH, CS_2_/ ethanol, reflux; (**d**) the appropriate alkyl halide, K_2_CO_3_/ acetone, r.t.

**Figure 3:**
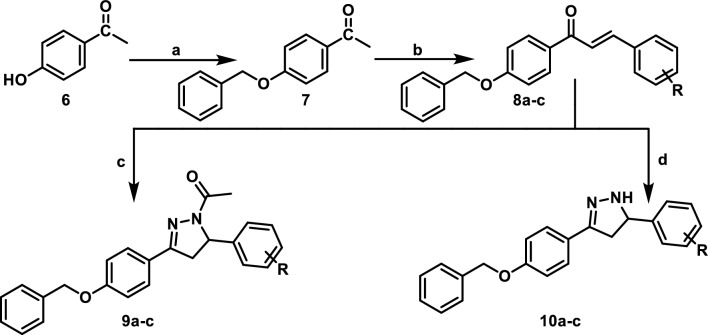
Synthesis of the target pyrazoline derivatives **9a-c** and **10a-c**. Reagents and Conditions: (**a**) benzyl chloride, K_2_CO_3_/DMF, r.t.; (**b**) the appropriate aldehyde, NaOH/ethanol, r.t.; (**c**) hydrazine hydrate/ glacial acetic acid, reflux; (**d**) hydrazine hydrate/ ethanol, reflux.

**Figure 4:**
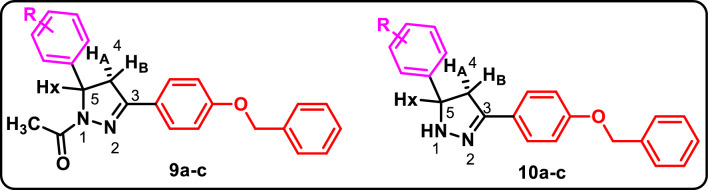
ABX spin system on the pyrazoline derivatives **9a-c** and** 10a-c**.

In Fig. 2, reaction of methyl 4-hydroxybenzoate **(1)** with benzyl chloride in dimethylformamide (DMF) in presence of potassium carbonate afforded methyl 4-(benzyloxy)benzoate **(2)**^28^. Refluxing of compound (**2**) with hydrazine hydrate in absolute ethanol yielded the corresponding hydrazide (**3**)^29^. Cyclization of the hydrazide using carbon disulfide in ethanol in presence of potassium hydroxide furnished compound (**4**)^29^ which was subjected to nucleophilic substitution with various alkyl halides affording compounds **5a-f** in a good yield. The ^1^H NMRspectra of compound **5a-c** showed singlet peak at (5.22–5.23 ppm) confirming the presence of (CH_2_) moiety of benzyl chloride derivatives. Meanwhile, compounds **5d** and **5e** showed three multiplet peaks at (1.55–4.06 ppm) corresponding to protons of cycloheptyl and cyclopentyl moiety. Furthermore, **5f.** showed triplet peak at 1.20 ppm, quartet peak at 4.17 ppm and singlet peak at 4.28 ppm corresponding to (**CH**_**3**_CH_2_), (CH_3_**CH**_**2**_) and (**CH**_**2**_COO) moiety of ethyl acetate. In Fig. 3, nucleophilic substitution of 4-hydroxyacetophenone **(6)** with benzyl chloride afforded 1-(4-(benzyloxy)phenyl)ethan-1-one **(7)**^30^. Through base catalyzed Claisen-Schmidt condensations of compound **(7)** with appropriate aldehyde, α, β-unsaturated carbonyl derivatives **8a-c** have been formed^31^. The ^1^H NMRspectra of **8c** showed two coupled doublet of vinylic protons at δ = 7.68 and δ = 7.85 ppm, with *J* = 15.5 Hz, indicating *trans*-configuration^32^. Cyclocondensation of chalcone intermediate **8a-c** using hydrazine hydrate in acetic acid as solvent^33^ yielded 1-(3-(4-(benzyloxy)phenyl)-5-(aryl)-4,5-dihydro-*1H*-pyrazol-1-yl)ethan-1-one derivatives **9a-c.** Also, cyclocondensation of **8a-c** using hydrazine hydrate in ethanol as solvent^34^ yielded 3-(4-(benzyloxy)phenyl)-5-(aryl)-4,5-dihydro-*1H*-pyrazole derivatives **10a-c.** The IR spectra of **9a-c** showed absorption bands at 1592–1617 cm^-1^ corresponding to C = N stretching bands because of ring closure while **10a-c** showed bands at 3500–3515 cm^-1^ due to NH functionality. The pyrazoline ring has a stereogenic carbon, thus compounds **9a-c** and **10a-c** exist in two stereoisomers R and S. This was obviously revealed by ^1^H NMR spectra which verified the assigned structures of **9a-c** and **10a-c** and showed clear characteristic signals of ABX spin system on the pyrazoline ring (Fig. 4)^35^. The two geminal protons of the pyrazoline ring resonated as two doublets of doublets at δ 3.11–3.23 ppm (H_4A_) and δ 3.79–3.88 ppm (H_4B_), with a geminal coupling constant of 18.0–18.1 Hz for **9a-c** and at δ 2.81–2.87 ppm (H_4A_) and δ 3.44–3.83 ppm (H_4B_), with *J*_*AB*_ = 16.4–16.1 Hz for **10a-c**. Conversely, in both series, the vicinal H_X_ pyrazoline proton showed up as a doublet of doublet at δ 4.47–5.70 ppm as a result of coupling with two magnetically non-equivalent protons, with coupling constants of 3.4–4.8 Hz for coupling with *trans* H_4A_ and 9.4–12.0 Hz for coupling with *cis* H_4B_. In addition, ^1^H NMR spectra of **9a-c** showed peaks δ 2.30–2.32 ppm belonging to the CH_3_ protons of the acetyl group. ^13^C NMR spectra of **9a-c** and **10a-c** offered further evidence for pyrazoline structure indicating the absence of trans-olefinic carbon signals and the presence of signals at δ 41.26–68 ppm corresponding for C_4_ pyrazoline and δ 55.90–63.12 ppm due to C_5_ of pyrazoline.

## Biological evaluation

### In vitro anti-proliferative screening

The newly synthesized compounds **5a-f, 9a-c** and **10a-c** were screened for their in vitro anti-proliferative activity via standard MTT assay^36,37^, using three human tumor cell lines namely; colorectal carcinoma HCT-116, hepatocellular carcinoma HEPG-2 and breast cancer MCF-7 cell lines in which EGFR is expressed^27^. Doxorubicin (DOX) was used as a reference drug. The results expressed as IC_50_ were summarized in Table 1.

**Table 1:** In vitro cytotoxic activities (IC_50_, µM and S.D values) of compounds **5a-f, 9a-c** and **10a-c** against HCT116, HePG-2, MCF7 and nonmalignant cells WI-38 cell lines. Signifcant values are in bold. ^a^IC_50_ value is the concentration required to produce 50% inhibition of cancer cell proliferation. Data are presented as the mean ± SD from the dose–response curves of three independent experiments.


Comp.no	R	IC_50_ (µM)^a^			
		HCT116	HePG-2	MCF7	WI-38
**5a**	benzyl	64.36 ± 3.7	**35.58 ± 2.2**	78.52 ± 4.1	53.87 ± 3.0
**5b**	4-Clbenzyl	73.15 ± 4.2	38.13 ± 2.2	84.26 ± 4.4	13.22 ± 1.1
**5c**	4-CH_3_benzyl	79.81 ± 4.4	71.38 ± 3.9	92.19 ± 4.9	> 100
**5d**	cycloheptyl	88.60 ± 4.8	82.79 ± 4.5	> 100	46.60 ± 2.7
**5e**	cyclopentyl	75.58 ± 4.2	59.33 ± 3.4	86.83 ± 4.5	11.71 ± 0.9
**5f.**	CH_2_COOCH_2_CH_3_	> 100	91.34 ± 5.0	> 100	77.31 ± 3.9
**9a**	3-Br	55.30 ± 3.1	32.58 ± 2.1	46.05 ± 2.5	85.13 ± 4.1
**9b**	3-NO_2_	**10.19 ± 0.9**	**7.83 ± 0.5**	**15.17 ± 1.2**	55.91 ± 3.2
**9c**	3,4-diOCH_3_	**16.18 ± 1.2**	**12.21 ± 0.9**	**8.53 ± 0.6**	49.61 ± 2.8
**10a**	3-Br	**9.94 ± 0.8**	**6.32 ± 0.4**	**8.11 ± 0.6**	62.31 ± 3.4
**10b**	3-NO_2_	**8.73 ± 0.7**	**3.98 ± 0.2**	**5.97 ± 0.3**	37.94 ± 2.3
**10c**	3,4-diOCH_3_	**5.55 ± 0.3**	**1.82 ± 0.1**	**2.86 ± 0.1**	41.17 ± 2.4
**DOX**	–	5.23 ± 0.3	4.50 ± 0.2	4.17 ± 0.2	6.72 ± 0.5

### Structure–activity correlation

As shown in Table 1**,** the pyrazole moiety proved to be essential for the cytotoxic activity, while the oxadiazole moiety in **5a-f** greatly caused deterioration of activity except for the benzyl derivative **5a** which exhibited moderate activity against HePG-2 with IC_50_ 35.58 μM. The nature of the substitution in 3-position of phenyl ring didn’t greatly affect the activity either electron donating or withdrawing. Compounds **9b,c** exhibited strong to high moderate activity against the three tested cell lines. Pyrazoline derivatives **10a-c** showed strong activity against the tested cell lines with compound **10c** showing the best activity with IC_50_ of 5.55, 1.82 and 2.86 μM against HCT-116, HePG-2 and MCF-7, respectively even better than the DOX the reference drug (IC_50_ = 5.23, 4.50, 4.17 μM, respectively) confirming the importance of the pyrazoline scaffold for the cytotoxic activity.

### In vitro cytotoxicity against human normal cell

The selectivity of the newly synthesized compounds was investigated on WI-38 normal fibroblast cells using Dox as reference drug (Table 1). It is interesting that, the investigated drugs have decreased cytotoxicity against normal fibroblast cells WI-38. The most active compounds in each series **5a, 9b, 10a, 10b** and **10c** showed low toxicity towards WI-38 with IC_50_ of 53.87, 55.91, 62.31, 37.94 and 41.17 μM, respectively compared to DOX (IC_50_ = 6.72 μM). This additional study will pave the path for more selective and less toxic EGFR inhibitors.

### EGFR assay

Using ELISA-based EGFR-TK assay, EGFR kinase activity was determined with gefitinib as the reference drug using different concentrations (0.01, 0.1, 1, 10, 100 µM) of the most active candidates in each series **5a, 9b, 10a, 10b** and **10c**. IC_50_ values were calculated and compared to the positive control, gefitinib (Table 2). It was observed that, compound **5a** and **10b** exhibited the best EGFR inhibition activity with IC_50_ values 0.09 and 0.16 µM, respectively in comparison to gefitinib (IC_50_ = 0.04 µM). In addition, compounds **9b** and **10c** showed moderate inhibitory activity with IC_50_ values 0.20 and 0.27 µM, respectively. On the other hand, compound **10a** displayed the weakest activity with IC_50_ values equal 0.70 µM. As a result, except for **10a**, the most cytotoxic compounds are good EGFR inhibitors in a dose-dependent manner.

**Table 2:** EGFR inhibition results (IC_50_ µM) of compounds **5a, 9b** and **10a-c** against gefitinib. Signifcant values are in bold.

Comp.no	EGFR IC_50_ (µM)	Comp.no	EGFR IC_50_ (µM)
5a	**0.09 ± 0.00**	**10b**	**0.16 ± 0.00**
9b	0.20 ± 0.01	**10c**	0.27 ± 0.01
10a	0.70 ± 0.03		
Gefitinib	0.04 ± 0.00		

In order to provide a more comprehensive understanding of the activity of compound **5a**, we have evaluated its specificity on other EGFR family members; HER‐2 (ErbB‐2), HER‐3 (ErbB‐3), and HER‐4 (ErbB‐4) relative to gefitinib as a positive control. As shown in Table 3, compound **5a** displayed nearly twice the activity of gefitinib against HER3. In addition, **5a** showed good inhibitory activity against HER4 (IC_50_ = 0.37 µM) compared to gefitinib (IC_50_ = 0.58 µM). On the contrary, **5a** had weaker inhibitory activity against HER2 (IC_50_ = 0.72 µM) than gefitinib (IC_50_ = 0.51 µM).

**Table 3:** HER‐2, HER‐3, and HER‐4 inhibition results (IC_50_ µM) of compound **5a** against gefitinib. Signifcant values are in bold.

Comp.no	HER2 IC_50_ (µM)	HER3 IC_50_ (µM)	HER4 IC_50_ (µM)
5a	0.72 ± 0.028	**0.18 ± 0.006**	0.37 ± 0.015
Gefitinib	0.51 ± 0.02	0.35 ± 0.012	0.58 ± 0.023

### Cell cycle analysis

**Figure 5:**
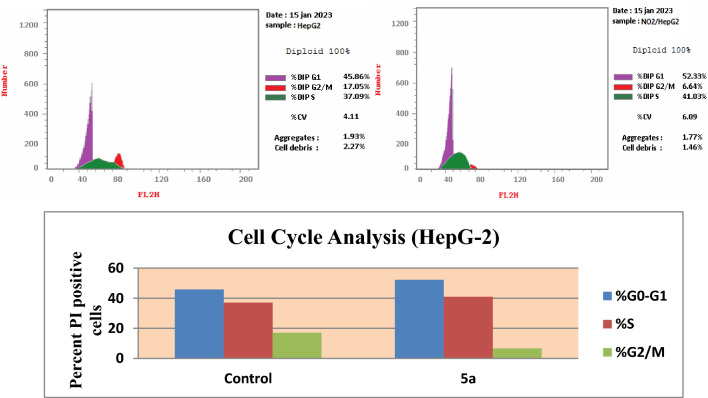
Effect of compound **5a** on DNA-ploidy flow cytometric analysis of HepG-2 cells. The cells were treated with DMSO as control and **5a** for 24 h.

In order to assist the mechanism of cell growth inhibition of compound **5a**, cell cycle dissemination and induction of apoptosis on HepG-2 cells was carried out using flow cytometry analysis^38^ by treating HepG-2 cancer cells with IC_50_ concentration of that compound for 24 h then stained with propidium iodide (PI) and analyzed by flow cytometry using BD FACSCalibur reader. HepG-2 cells were chosen because compound **5a** showed the highest antitumor activity against HepG-2 cell line rather than the other cell lines (IC_50_ = 35.58 µM). The PI staining data (Table 4 and Fig. 5) showed that treatment with compound **5a** resulted in a significant increase in the ratio of HepG-2 cells in the G0/G1 phase from 45.86% (untreated cells) to 52.33% and in the S phase from 37.09% (untreated cells) to 41.03% with a concomitant decrease in the number of cells in G2/M phase by 6.64% compared to untreated control (17.05%). These results indicate that compound **5a** arrested the cell cycle on HepG-2 at a G1/S phase.

**Table 4:** Cell cycle analysis results for compound **5a** on HepG-2 cells^a^. ^a^The data are present as the average of at least three independent experiments.

Comp.no	%G0-G1	%S	%G2/M	Comment
5a	52.33	41.03	6.64	cell growth arrest at G1/S
Control	45.86	37.09	17.05

### Apoptosis mechanistic studies

Anti-cancer drugs act through different signaling pathways eventually converge to the onset of cell death through apoptosis. The ability of tumor cells to avoid apoptosis is a hallmark of human cancer. In expansion to cancer cell survival, absconds in apoptotic pathways may moreover contribute to tumor movement and chemo-resistance^39^. Hence, focusing on apoptosis may be a bull's eye inclining approach within the revelation and improvement of novel anticancer therapeutics. Hence, it was considered of intrigued to examine the apoptosis actuating impact of compound **5a** on HepG-2 cells.

### AnnexinV-FITC/Propidium iodide dual staining assay

**Figure 6:**
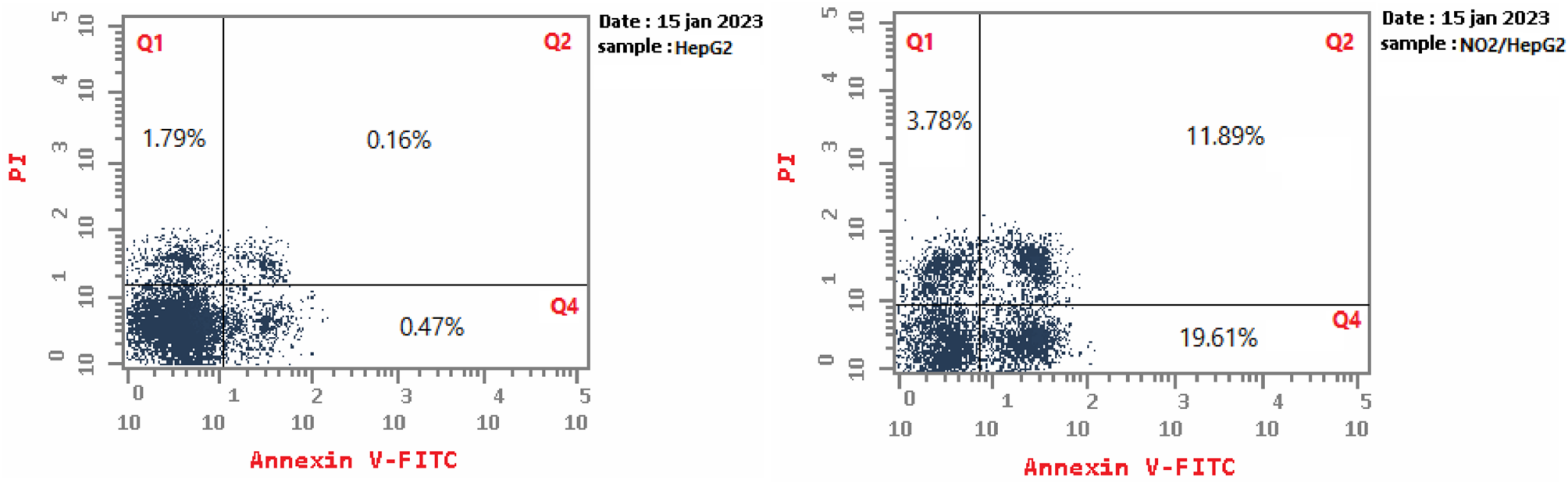
Effect of compound **5a** on the percentage of annexin V-FITC-positive staining in HepG-2 cells. The cells were treated with DMSO as control and **5a** for 24 h. Q1 quadrant represents dead (necrotic) cells; Q2 quadrant represents late apoptosis; Q3 quadrant represents live cells; Q4 quadrant represents early apoptosis.

**Figure 7:**
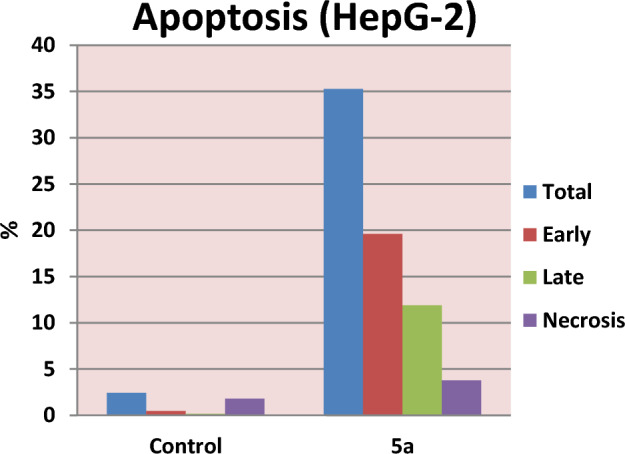
Apoptosis analysis for compound **5a**.

Liver HepG-2 cancer cells were used in the Annexin V-FITC/Propidium iodide dual staining assay^40^ to calculate the percentage of apoptosis induced by compound **5a**. As shown in Figs. 6 and Fig. 7, compound **5a** induces the early apoptosis in HepG-2 after 24 h incubation by (19.61%) 41.72 folds over the untreated cells (0.47%). Also, it enhances the late apoptotic induction by 11.89% compared to untreated control (0.16%). Cumulatively, compound **5a** induced total apoptosis (35.28%) 14.57 folds over the untreated cells (2.42%).

### Effect of compound 5a on mitochondrial apoptosis using gene expression analysis (RNA extraction and real-time RT-PCR for tested genes)

**Figure 8:**
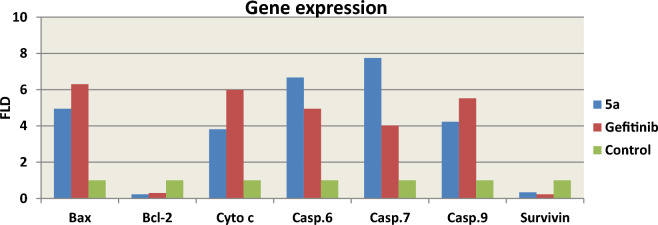
RT-PCR data for gene expression of BAX, BCL-2, Cyto c, Casp. 6, 7, 9 and Survivin in HepG-2 cells treated with compound **5a**.

**Figure 9:**
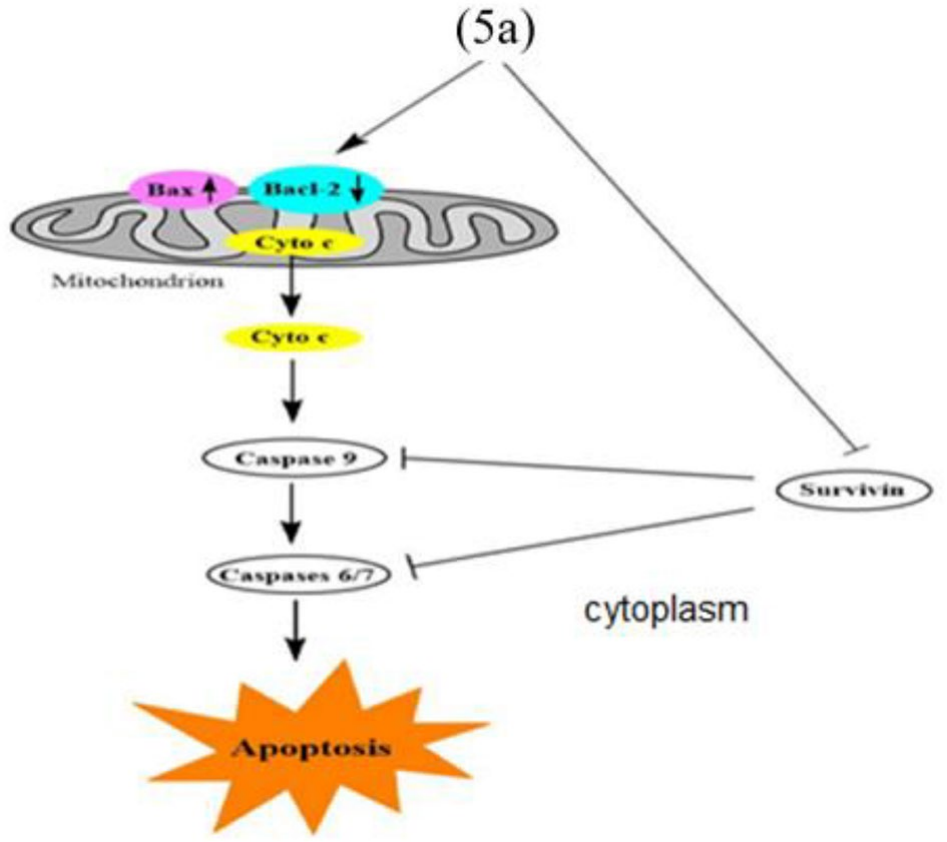
Mitochondrial-mediated apoptotic pathway induced by **5a** on HepG-2 cells.

The B cell lymphoma-2 (Bcl-2) family of proteins, which includes pro-apoptotic (Bax, Bak, Bid, and Bad) and anti-apoptotic (Bcl-2, Bcl-xL, and Bcl-w) proteins, mediates the mitochondrial or intrinsic apoptosis pathway. These proteins cause apoptosis by influencing the permeability of the mitochondrial membrane, which results in the release of cytochrome c into the cytosol^41^. The Bax/Bcl-2 ratio is frequently employed as a marker of cell death and is essential for controlling mitochondrial cytochrome c release^42^. It is thought that caspases perform critical functions in modulating the mitochondrial pathway. After cytochrome c is released into the cytosol, caspase-9 is triggered. Caspase-9 can activate effector caspases, such as caspase-3, 6 and 7, which cause cells to enter apoptosis^43^. The various target proteins that the effector caspases cleave are widely dispersed throughout the cell, which causes the morphological modifications that are indicative of apoptosis^44^. Apoptosis is inhibited by the anti-apoptotic protein survivin, which regulates the actions of caspase-9 and effector caspases^45^. Based on that, we looked into how compound **5a** might impact the expression of apoptotic and antiapoptotic markers in HepG-2 cells in order to induce intrinsic apoptosis using rotorgene RT- PCR system. According to Fig. 8, compound **5a** boosted the level of the proapoptotic protein; Bax by 4.95 folds and significantly decreased the levels of the anti-apoptotic protein; Bcl-2, compared to the control, raising the Bax/Bcl-2 ratio. Additionally, compared to control cells, compound **5a** increased the level of cytochrome c by 3.82 folds. Moreover, it increased level of caspases 6, 7 and 9 by 6.67, 7.57 and 4.23 folds, respectively, compared to the control. It also down regulated expression level of the antiapoptotic marker; survivin compared to the control cells. In brief, up regulation of caspases 6, 7 and 9 besides elevated Bax/ Bcl-2 ratio suggests that compound **5a** induces apoptosis in human HepG-2 cells through mitochondrial-mediated pathway (Table 5, Fig. 9).

**Table 5:** RT-PCR gene expression of BAX, BCL-2, Cyto c, Casp. 6, 7, 9 and Survivin for compound **5a** in HepG-2 cells.

Compound	RT-PCR Fold Change						
	Bax	Bcl-2	Cyto c	Casp. 6	Casp. 7	Casp. 9	Survivin
**5a**	4.95	0.23	3.82	6.67	7.57	4.23	0.34
**Gefitinib**	6.30	0.30	5.99	4.95	4.02	5.52	0.23
**Control**	1	1	1	1	1	1	1

### Intracellular ROS accumulation assay

**Figure 10:**
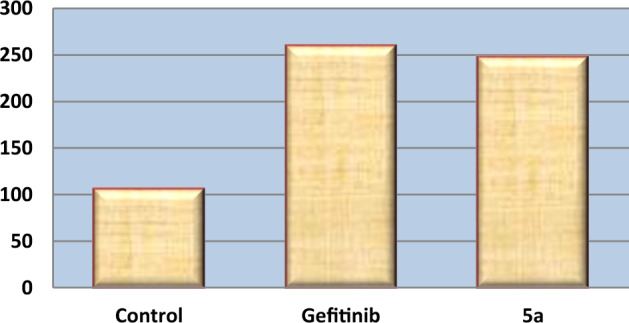
Effect of **5a** on intracellular ROS accumulation in HepG-2 cells.

Under both physiological and pathological circumstances, reactive oxygen species (ROS) such H_2_O_2_ and superoxides play a significant role in the activation of apoptosis. It's interesting to note that mitochondria both produce and absorb ROS. Direct or indirect ROS activity appears to be a major mediator of cytochrome c release from mitochondria, which initiates caspase activation^46^ so, investigating compound **5a**'s impact on the ROS-apoptotic pathway proved intriguing. The intracellular ROS concentration was detected by using ELISA-technique via comparing the concentration of ROS in cells treated by compound **5a** with the control untreated HepG-2 cells. As illustrated in Table 6 and Fig. 10, compound **5a** stimulates ROS accumulation (247.8 Pg/ml) in HepG-2 cells slightly lower than gefitinib (260 Pg/ml) and 2.34 folds higher than the control cells. As depicted from the results, compound **5a** caused the mitochondrial pathway ROS-mediated apoptosis in HepG-2 cells.

**Table 6:** Effect of **5a** and gefitinib on intracellular ROS accumulation in HepG-2 cells. Values were reported as mean ± SD of three independent experiments.

Comp.no	ROS (Pg/ml)	Fold
5a	247.8 ± 31.1	2.34
Gefitinib	260 ± 14.7	2.46
Control	105.6 ± 10.8	1

### Molecular modeling study

The use of molecular docking has proven to be an effective method in drug discovery for understanding how small molecules interact with different biological targets. This provides an opportunity to enhance and create more effective therapeutic drugs^47,48^. By studying the inhibition affinity of gefitinib with the EGFR binding site, it was found that the quinazoline moiety fits into the ATP binding pocket in the kinase domain forming H-bond with hinge region due to N-1 and N-3 atoms. In addition, large aniline substituent and 6-morpholinylpropoxy group filled the hydrophobic pocket and the solvent region; respectively^49,50^. The molecular docking study showed that compound **5a** and **10b** showed the best binding affinity while compound **10a** showed the lowest in comparison to the standard inhibitor gefitinib as shown in Table 7**,** which agrees with the experimental data.

**Table 7:** Binding affinity of compounds **5a, 10a** and **10b** under investigation and gefitinib.

Comp.no	Affinity (kcal/mol)	EGFR IC_50_ (µM)
5a	−8.1	0.09 ± 0.00
10a	−7.3	0.70 ± 0.03
10b	−7.9	0.16 ± 0.00
Gefitinib	−8.6	0.04 ± 0.00

**Figure 11:**
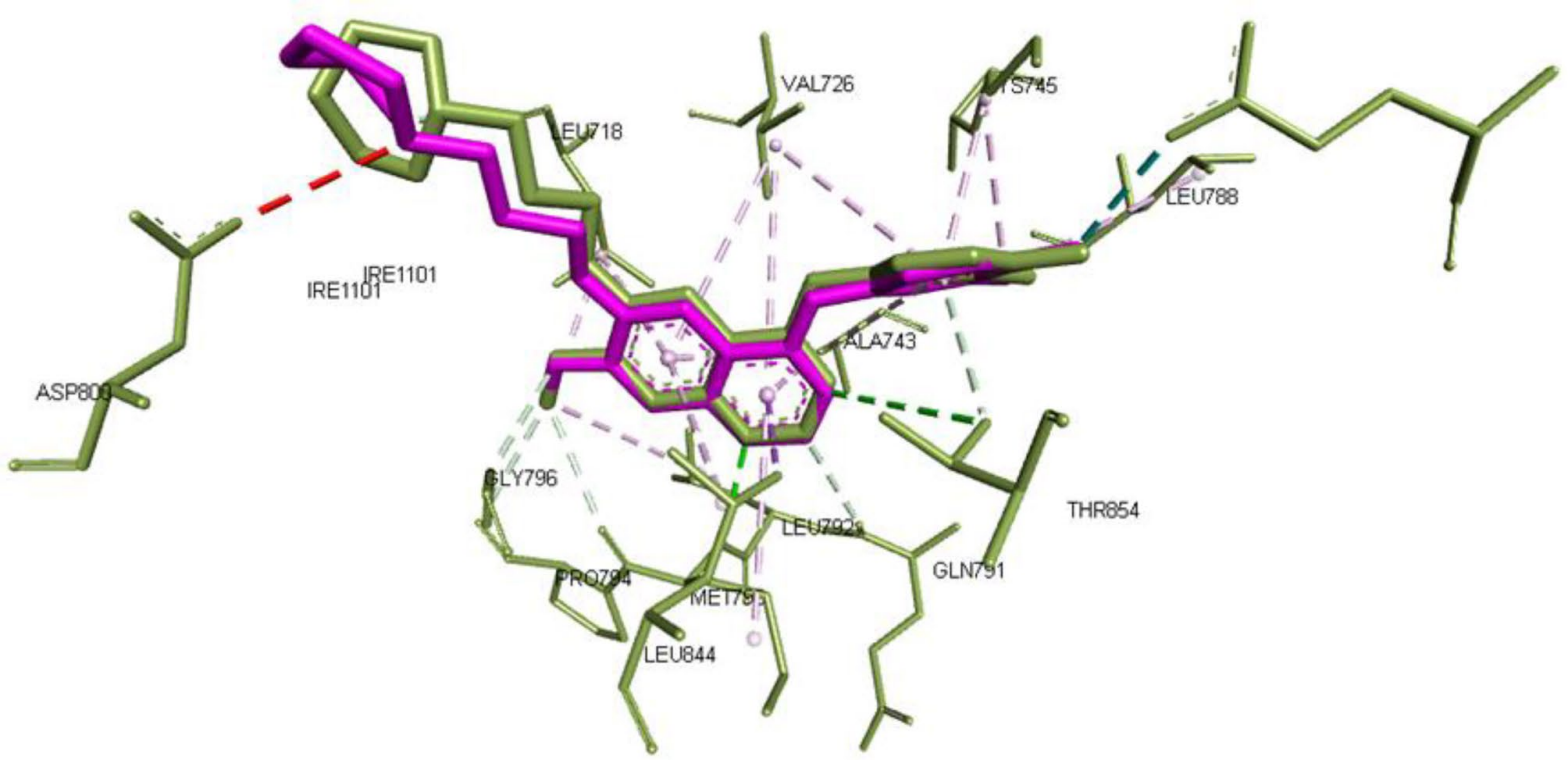
Redocking of gefitinib in the active site of EGFR where Experimental pose is presented as green sticks and the redocked pose as magenta with RMSD = 0.6.

Firstly, the redocked posed of gefitinib to the active site using PyRX software was similar to that observed in the experimentally produced pose in X-ray crystallography with RMSD = 0.6 implying the validity of the used software. Gefitinib was able to interact with Met793, Lys745 and Gln791 through hydrogen bonding and Leu718, Gly719, Asp800, Leu844, Thr854, Pro794, Phe795 through hydrophobic interaction. This is in accordance with the reported binding mode of gefitinib^51^ (Fig. 11).

**Figure 12:**
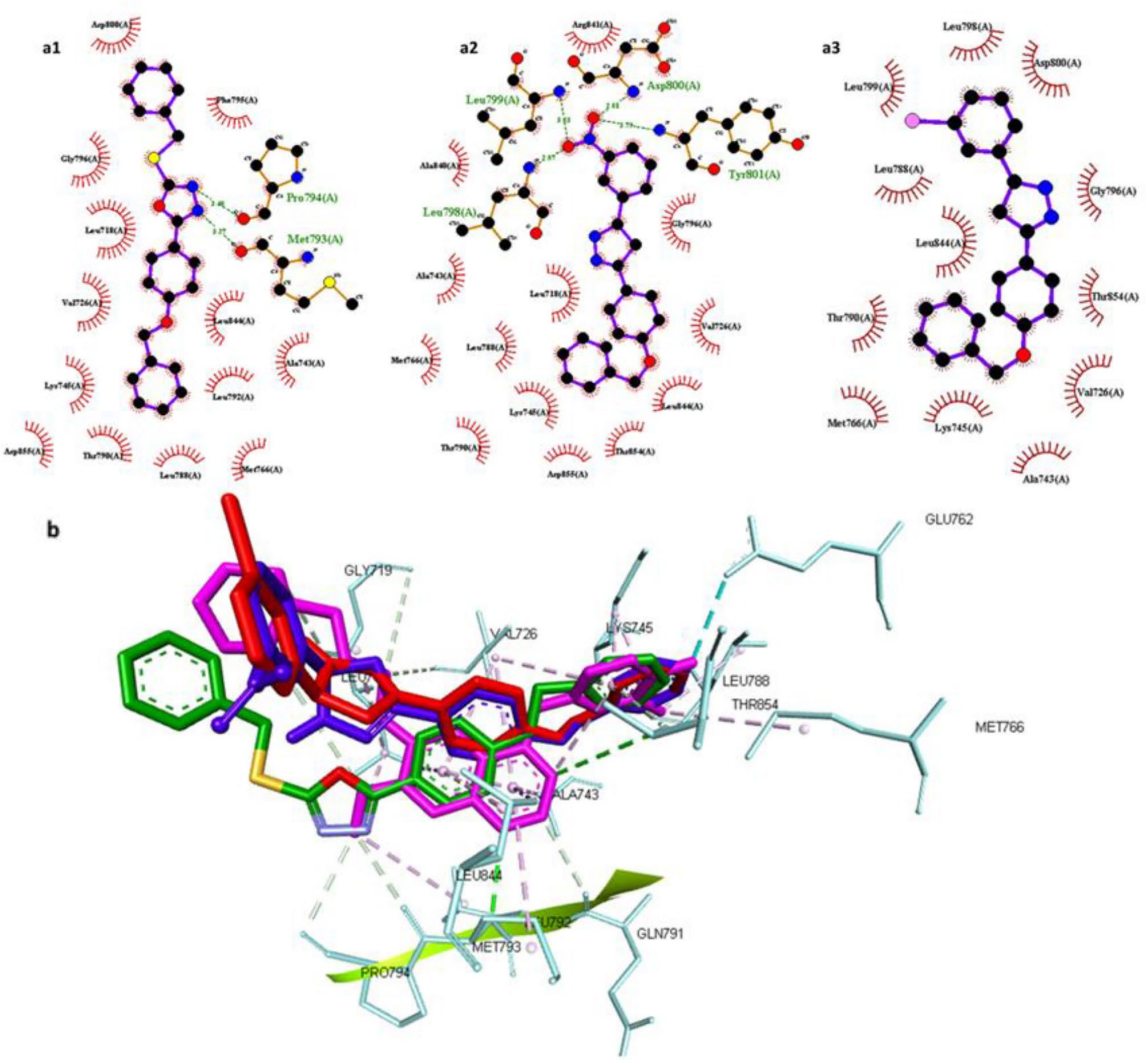
(**a**) 2D interaction of compound **5a** (a1) **10b** (a2) **10a** (a3) with EGFR active site where direct bonds are shown as dashed lines while hydrophobic interactions are shown as spline sections. B) 3D representation of cluster of the docked pose of **5a** (Green), **10b** (Blue) **10a** (red) overlied to the co-crystallized ligand gefitinib (Magenta).

Meanwhile, Compound **5a** showed similar binding mode to gefitinib as N-3 and N-4 of the oxadiazole ring was able to interact with key amino acids Met793 and Pro794 through hydrogen bonding with bond length 3.27 and 3.40 ^◦^A, respectively. Interestingly, the sulfanyl moiety was oriented towards the solvent accessible pocket leading to interaction with ASP800. The 4-(benzyloxy)phenyl) moiety was able to exert some hydrophobic interactions with the back pocket of ATP such as Leu718, Val726, Leu745, Leu844, Thr790 and Leu792 as depicted in Fig. 12a-1 which could explain the good enzyme inhibition activity achieved by this compound in comparison to the standard gefitinib^51^.

In case of Compound **10b,** the reduced affinity shown in experimental enzyme inhibition assay was explained by its inability to form hydrogen bonds with residue in the ATP active site. Yet, this was compensated by the ability of the nitro group to form extensive hydrogen bonding with Leu798, Leu799, Asp800, and Tyr801, also the 4-(benzyloxy)phenyl) moiety was able to exert similar interactions like **5a** as shown in Fig. 12a-2.

Finally, the molecular docking of **10a** revealed the dramatic decrease in the affinity to EGFR where it was not able to form any hydrogen bonds with the active site but other hydrophobic interaction with Lys754, Leu789, Thr 790, Leu799, Gly796, Asp800, Leu844, Thr854 as shown in Fig. 12a-3. The 3D alignment of compounds under investigation and gefitinib showed that the better activity of **5a** is attributed to the ability of oxadiazole ring to form hydrogen bond with Met793 which was crucial to produce interaction similar to that exhibited by gefitinib, this is supported by the inability of 4,5-dihydropyrazole in **10b** and **10a** to interact with such key residue leading to the protrusion of the whole molecule towards the solvent accessible pocket instead of the ATP pocket leading to the observed decrease in the activity as shown in Fig. 12b.

### In silico* studies*

#### Evaluation of physicochemical, and pharmacokinetic prediction on active compounds

The Lipinski rule of five compliance of the two most potent newly synthesized compounds **5a** and **10b** was examined using Swiss ADME: a free web tool^52^ as illustrated in Table 8. The obtained results revealed that compound **5a** has one violation with slightly increase in MLOGP (4.19) > 4.15) while compound **10b** is in full accordance to Lipinski's rule of five. Number of rotatable bonds of all investigated compounds was ≤ 10, indicating acceptable molecular flexibility with consequent expected good permeability and oral bioavailability.

**Table 8:** In silico the physicochemical and pharmacokinetic properties of compounds **5a** and **10b** as well as standard drug (Gefitinib).* MW; Molecular weight, nHBA; no. of hydrogen bond acceptor, nHBD; no of hydrogen bond donor, MLogP; an octanol–water partition coefficient, nRB; no. of rotatable bond, TPSA; topological polar surface area.

Comp.no	Lipinski’s Rule					Veber filter		Pharmacokinetics	
	MW ≤ 500	nHBA ≤ 10	nHBD ≤ 5	MLOGP ≤ 4.15	nVs ≤ 1	nRB ≤ 10	TPSA ≤ 140 Å^2^	GI	BBB
**5a**	374.46	4	0	4.19	1	7	73.45	High	No
**10b**	373.41	4	1	3.60	0	6	79.44	High	No
Gefitinib	446.90	7	1	2.82	0	8	68.74	High	Yes

By the same way, some Pharmacokinetic properties were studies depending on Swiss ADME: a free web tool involving gastrointestinal absorption and blood brain barrier penetration. The two investigated compounds **5a** and **10b** showed high gastrointestinal absorption as gefitinib but unlike it, they showed no permeation to blood brain barrier.

#### Bioavailability radar

**Figure 13:**
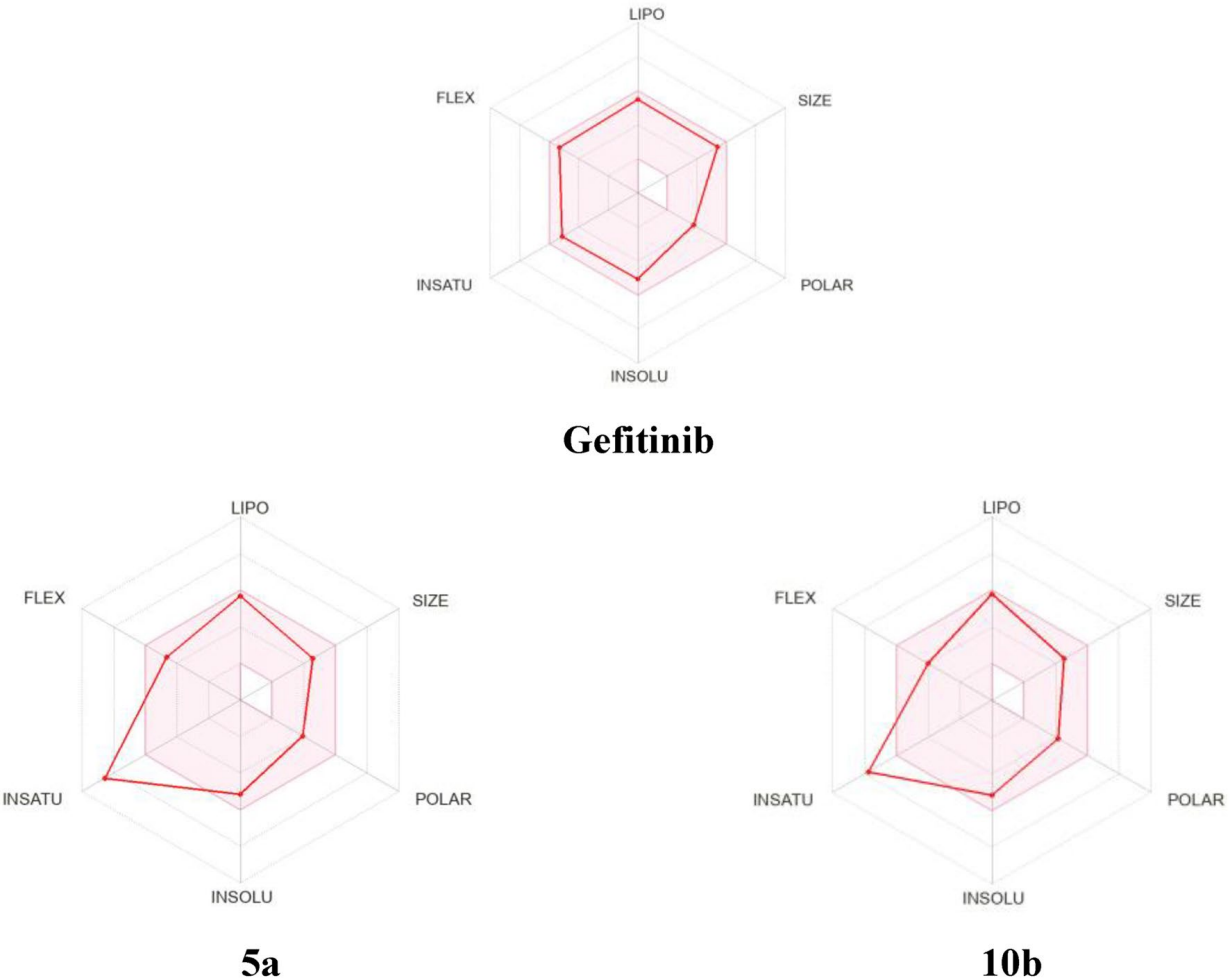
Bioavailability radar for gefitinib and the most active compounds.

For a quick assessment of drug-likeness, the bioavailability radar is provided. It was acquired with the help of the SwissADME web database^52^. The next six physicochemical characteristics: lipophilicity, size, polarity, solubility, flexibility, and saturation are considered. These requirements are crucial for a drug similarity molecule. The ideal range for each property is represented by the pink area. Size: MW between 150 and 500 g/mol, polarity: TPSA between 20 and 130, insolubility: expressed by logS (ESOL): not more than 0, insaturation: fraction of sp3 hybridized carbons not less than 0.25, flexibility: not more than 9 rotatable bonds, and lipophilicity represented by XLOGP3 between -0.7 and + 5.0. Figure 13 represented the obtained results which were in accordance with the results in Table 6. The values imply that compounds **5a** and **10b** fell in the pink area except for insaturation which is 0.09 and 0.14, respectively.

Finally, our new compounds exhibited not only promising activity, but also some oral bioavailability beside pharmacokinetic properties as gefitinib.

## Conclusion

Two new series of oxadiazole and pyrazoline derivatives were designed, synthesized and evaluated for their anti-proliferative activity against three human cell lines; colorectal carcinoma (HCT-116), hepatocellular carcinoma (HepG-2) and breast carcinoma (MCF-7). On WI-38 normal fibroblast cells, the newly synthesized compounds' selectivity towards normal and cancer cells was studied. The most effective candidates in each series included compounds **5a, 9b**, **10a, 10b**, and **10c**, which exhibited strong cytotoxic activity against all three cancer cell lines while being highly selective for cancer cells. They were then tested for their ability to inhibit the EGFR enzyme, with compound **5a** and **10b** exhibiting the best inhibition activity with IC_50_ values 0.09 and 0.16, respectively in comparable to gefitinib (IC_50_ = 0.04 µM). Compound **5a** displayed good activities against HER3 and HER4 with IC_50_ values 0.18 and 0.37 µM, respectively compared to gefitinib (IC_50_ = 0.35 and 0.58 µM, respectively). The most active **5a** induced both cell cycle arrest at G1/S phase in HepG-2 cells and a significant increase in the total apoptosis via raising the Bax/Bcl-2 ratio, increasing the level of cytochrome C. Furthermore, it activated caspases 6, 7 and 9 and down regulated expression level of the antiapoptotic marker; surviving. It also increased accumulation of ROS in HepG-2 cells. Comparative molecular docking study was performed between the most potent EGFR inhibitors **5a** and **10b**, and the least potent one **8d**, where compounds **5a** and **10b** showed a good binding with EGFR catalytic active site via the key amino acids that confirmed their higher EGFR inhibitory activity. Contrarily, compound **10a**, which was the least effective EGFR inhibitor, lacked interaction with important pocket residues, which explained its low enzyme activity. Lipinski’s rule of five beside some pharmaceutical properties were calculated where compounds **5a** and **10b** represented very promising orally absorbed candidates due to their reasonable drug-likeness with acceptable physicochemical properties.

### Experimental

#### General

Synthesis of the target compounds was done at laboratory of pharmaceutical organic department, faculty of pharmacy, Mansoura University, Egypt. Using thin layer chromatography technique on silica gel plates 60 F245 (E. Merck, Germany), reaction times were determined. Detection and visualization of spots were detected by UV light (366, 245 nm). Melting points were determined on Stuart melting point apparatus and are uncorrected. Microanalyses were performed at Cairo University and performed on a Perkin-Elmer 240 elemental analyzer for C, H and N elements and the results were within the acceptable range of the theoretical values. ^1^H and ^13^C NMR were performed at Mansoura University and recorded on a JEOL 500 MHz FT spectrometer, Brucker 400 MHz spectrometer and 125 MHz spectrometer, respectively. Chemical shifts are expressed in *δ* ppm with reference to TMS. Using a Nicolet iS10 infrared spectrometer, IR spectra were recorded at Mansoura University. Mass spectra were carried out on Direct Inlet part to mass analyzer in Thermo Scientific GCMS model ISQ at the Regional Center for Mycology and Biotechnology (RCMB), Al-Azhar University, Nasr City, Cairo. The HRMS analysis was recorded on LC/Q-TOF, 6530 (Agilent Technologies, Santa Clara, CA, USA) equipped with an autosampler (G7129A), a quat. pump (G7104C) and a column comp (G7116A) at faculty of pharmacy, Fayoum University. All the used chemicals and reagents were purchased from Aldrich Chemicals Co, USA and commercial sources. Molecular modeling experiments were performed using Autodock vina as the docking engine. Lipinski's rule of five, ADMET and the bioavailability radar were obtained by the aid of online SwissADME database. Antitumor screening of all the newly synthesized compounds, the in vitro EGFR inhibition assay, cell cycle analysis and apoptosis assay were performed at Holding company for biological products and vaccines (VACSERA), Cairo, Egypt.

### Chemistry

#### General procedure for the synthesis of compounds 5a-f

A mixture of 5-(4-(benzyloxy)phenyl)-1,3,4-oxadiazole-2-thiol **(4)** (0.5 g, 0.002 mol), the appropriate alkyl halide derivative (0.002 mol) and potassium carbonate (0.55 g, 0.004 mol) in acetone was stirred for 18 h. The progress of the reaction was monitored by TLC. After the reaction was complete, it was poured on iced water; the precipitate was filtered, washed with water, dried and crystallized from aqueous ethanol.

##### *2-(4-(Benzyloxy)phenyl)-5-(benzylthio)-1,3,4-oxadiazole* (5a)

Yield (90%), m.p. 118–120^◦^C. IR (KBr, cm^−1^): 1680 (C = N), 1574 (C = C). ^1^H NMR (DMSO d6, 400 MHz): δ 4.58 (s, 2H, CH_2_), 5.22 (s, 2H, CH_2_), 7.23 (d, *J* = 8.9 Hz, 2H, Ar–H), 7.28–7.50 (m, 10H, Ar–H), 7.91 (d, *J* = 8.8 Hz, 2H, Ar–H). ^13^C NMR (DMSO d6): δ 36.43, 70.03, 116.01, 116.18, 128.24, 128.31, 128.53, 128.76, 128.99, 129.06, 129.50, 136.92, 137.12, 161.64, 162.96, 165.65. HRMS (ESI): m/z calcd for C_22_H_18_N_2_O_2_S: 375.11672 [M + H]^+^. Found 375.11704. Anal. Calcd for C_22_H_18_N_2_O_2_S (374.46): C, 50.75; H, 4.85; N, 7.48; %. Found: C, 50.70; H, 4.86; N, 7.50%.

##### *2-(4-(Benzyloxy)phenyl)-5-((4-chlorobenzyl)thio)-1,3,4-oxadiazole* (5b)

Yield (91%), m.p. 125–127^◦^C. IR (KBr, cm^−1^): 1640 (C = N). ^1^H NMR (DMSO d6, 400 MHz): δ 4.57 (s, 2H, CH_2_), 5.21 (s, 2H, CH_2_), 7.22 (d, *J* = 8.8 Hz, 2H, Ar–H), 7.36–7.52 (m, 9H, Ar–H), 7.90 (d, *J* = 8.7 Hz, 2H, Ar–H). ^13^C NMR (DMSO d6): δ 35.57, 70.02, 115.98, 116.17, 128.31, 128.53, 128.77, 128.99, 129.01, 131.40, 132.85, 136.45, 136.92, 161.65, 162.79, 165.71. MS m/z (%): 408.60 (M^+^), 410.79 (M^+^ + 2). Anal. Calcd for C_22_H_17_ClN_2_O_2_S (408.90): C, 64.62; H, 4.19; N, 6.85; %. Found: C, 64.60; H, 4.20; N, 6.80%.

##### *2-(4-(Benzyloxy)phenyl)-5-((4-methylbenzyl)thio)-1,3,4-oxadiazole* (5c)

Yield (89%), m.p. 130–132^◦^C. IR (KBr, cm^−1^): 1639 (C = N). ^1^H NMR (DMSO d6, 400 MHz): δ 2.28 (s, 3H, CH_3_), 4.53 (s, 2H, CH_2_), 5.23 (s, 2H, CH_2_), 7.16–7.48 (m, 11H, Ar–H), 7.92 (d, *J* = 8.7 Hz, 2H, Ar–H). ^13^C NMR (DMSO d6): δ 21.18, 35.43, 70.03, 115.88, 116.97, 128.24, 128.31, 128.53, 128.76, 128.99, 129.06, 129.50, 136.92, 137.12, 161.64, 162.96, 165.65. MS m/z (%): 388.02 (M^+^). Anal. Calcd for C_23_H_20_N_2_O_2_S (388.49): C, 71.11; H, 5.19; N, 7.21; %. Found: C, 71.15; H, 5.17; N, 7.23%.

##### *2-(4-(Benzyloxy)phenyl)-5-(cycloheptylthio)-1,3,4-oxadiazole* (5d)

Yield (85%), m.p. 83–85^◦^C. IR (KBr, cm^−1^): 1640 (C = N). ^1^H NMR (DMSO d6, 400 MHz): δ 1.55–1.77 (m, 10H, cycloheptyl H), 2.12–2.17 (m, 2H, cycloheptyl H), 3.90–3.96 (m, 1H, cycloheptyl H), 5.22 (s, 2H, CH_2_), 7.23 (d, *J* = 8.8 Hz, 2H, Ar–H), 7.36–7.50 (m, 5H, Ar–H), 7.92 (d, *J* = 8.7 Hz, 2H, Ar–H). ^13^C NMR (DMSO d6): δ 25.53, 28.07, 34.91, 48.67, 70.01, 116.18, 128.30, 128.52, 128.76, 128.99, 136.92, 161.60, 163.02, 165.51, 167.85. MS m/z (%): 380.4 (M^+^). Anal. Calcd for C_22_H_24_N_2_O_2_S (380.51): C, 69.44; H, 6.36; N, 7.36; %. Found: C, 69.40; H, 6.30; N, 7.40%.

##### *2-(4-(Benzyloxy)phenyl)-5-(cyclopentylthio)-1,3,4-oxadiazole* (5e)

Yield (83%), m.p. 91–93^◦^C. IR (KBr, cm^−1^): 1635 (C = N). ^1^H NMR (DMSO d6, 400 MHz): δ 1.63–1.77 (m, 6H, cyclopentyl H), 2.18–2.25 (m, 2H, cyclopentyl H), 4.00–4.06 (m, 1H, cyclopentyl H), 5.22 (s, 2H, CH_2_), 7.23 (d, *J* = 8.8 Hz, 2H, Ar–H), 7.34–7.50 (m, 5H, Ar–H), 7.92 (d, *J* = 8.8 Hz, 2H, Ar–H). ^13^C NMR (DMSO d6): δ 24.77, 33.75, 46.07, 70.01, 116.10, 116.16, 128.30, 128.52, 128.74, 128.98, 136.92, 161.59, 163.26, 165.44. MS m/z (%): 352.6 (M^+^). Anal. Calcd for C_20_H_20_N_2_O_2_S (352.45): C, 68.16; H, 5.72; N, 7.95; %. Found: C, 68.20; H, 5.72; N, 7.90%.

##### *Ethyl 2-((5-(4-(benzyloxy)phenyl)-1,3,4-oxadiazol-2-yl)thio)acetate* (5f)

Yield (93%), m.p. 100–102^◦^C. IR (KBr, cm^−1^): 1637 (C = N). ^1^H NMR (DMSO d6, 400 MHz): δ 1.20 (t, *J* = 7.1 Hz, 3H, CH_3_), 4.17 (q, *J* = 7.1 Hz, 2H, CH_2_CH_3_), 4.28 (s, 2H, CH_2_CO), 5.22 (s, 2H, CH_2_), 7.23 (d, *J* = 8.7 Hz, 2H, Ar–H), 7.35–7.50 (m, 5H, Ar–H), 7.91 (d, *J* = 8.7 Hz, 2H, Ar–H). ^13^C NMR (DMSO d6): δ 14.45, 34.33, 62.06, 70.04, 115.93, 116.20, 128.33, 128.53, 128.75, 128.99, 136.91, 161.68, 162.63, 165.63, 168.25. MS m/z (%): 370.9 (M^+^). Anal. Calcd for C_19_H_18_N_2_O_4_S (370.42): C, 61.61; H, 4.90; N, 7.56; %. Found: C, 61.60; H, 4.87; N, 7.50%.

#### General procedure for synthesis of compounds 8a-c

A mixture of 1-(4-(benzyloxy)phenyl)ethan-1-one **(7)** (2.26 g, 0.01 mol), appropriate aldehyde (0.01 mol) and sodium hydroxide (1 g, 0.025 mol) in ethanol (20 ml) was stirred at room temperature for 2 h, the precipitate was filtered and washed with cold water then crystallization from ethanol.

##### *(E)-1-(4-(Benzyloxy)*phenyl*)-3-(3-bromophenyl)prop-2-en-1-one* (8a)

Yield (85%), m.p. 132–134^◦^C. IR (KBr, cm^−1^): 1658 (C = O). ^1^H NMR (DMSO-d6, 400 MHz): δ 5.26 (s, 2H, CH_2_), 7.19 (d, *J* = 8.7 Hz, 2H, Ar–H), 7.37–7.51 (m, 5H, Ar–H), 7.63–7.70 (m, 4H, Ar–H and olefenic H), 7.87 (d, *J* = 7.7 Hz, 1H, Ar–H), 8.05 (d, *J* = 15.6 Hz, 1H, olefenic H), 8.22 (d, *J* = 8.4 Hz, 2H, Ar–H).

##### *(E)-1-(4-(Benzyloxy)*phenyl*)-3-(3-nitrophenyl)prop-2-en-1-one* (8b)

Yield (87%), m.p. 156–158^◦^C. IR (KBr, cm^−1^): 1650 (C = O) .^1^H NMR (DMSO-d6, 400 MHz): δ 5.26 (s, 2H, CH_2_), 7.20 (d, *J* = 8.2 Hz, 2H, Ar–H), 7.36–7.51 (m, 5H, Ar–H), 7.76 (t, *J* = 8.0 Hz, 1H, Ar–H), 7.84 (d, *J* = 15.6 Hz, 1H, olefenic H), 8.22–8.36 (m, 5H, Ar–H and olefenic H), 8.79 (s, 1H, Ar–H).

##### *(E)-1-(4-(benzyloxy)phenyl)-3-(3,4-dimethoxyphenyl)prop-2-en-1-one* (8c)

Yield (81%), m.p. 109–111^◦^C. IR (KBr, cm^−1^): 1656 (C = O). ^1^H NMR (DMSO-d6, 400 MHz): δ 3.83 (s, 3H, OCH_3_), 3.88 (s, 3H, OCH_3_), 5.26 (s, 2H, CH_2_), 7.03 (d, *J* = 8.3 Hz, 1H, Ar–H), 7.18 (d, *J* = 8.7 Hz, 2H, Ar–H), 7.35–7.45 (m, 4H, Ar–H), 7.50 (d, *J* = 7.2 Hz, 2H, Ar–H), 7.55 (s, 1H, Ar–H), 7.68 (d, *J* = 15.5 Hz, 1H, olefenic H), 7.85 (d, *J* = 15.5 Hz, 1H, olefenic H), 8.18 (d, *J* = 8.7 Hz, 2H, Ar–H).

#### *General procedure for synthesis of compounds* 9a-c.

A mixture of compounds **(8a-c)** (0.001 mol) and hydrazine hydrate (99%, 0.25 ml, 0.002 mol) in glacial acetic acid (15 ml) was heated under reflux for 6–9 h. The mixture was cooled and poured onto crushed ice to yield the product which was extracted using ethylacetate (3 × 15 ml), dried over anhydrous sodium sulfate. The obtained solid after concentration was filtered and crystallized from ethanol.

##### *1-(3-(4-(Benzyloxy)*phenyl*)-5-(3-bromophenyl)-4,5-dihydro-1H-pyrazol-1-yl)ethan-1-one* (9a)

Yield (56%), m.p. 93–95^◦^C. IR (KBr, cm^−1^): 1658 (C = O), 1592 (C = N). ^1^H NMR (DMSO d6, 400 MHz): δ 2.31 (s, 3H, CH_3_), 3.15 (dd, *J* = 18.0, 4.2 Hz, 1H, pyrazoline H_4A_), 3.81 (dd, *J* = 18.0, 12.0 Hz, 1H, pyrazoline H_4B_), 5.17 (s, 2H, CH_2_), 5.52 (dd, *J* = 11.5, 4.1 Hz 1H, pyrazoline H_X_), 7.13 (d, *J* = 8.4 Hz, 2H, Ar–H), 7.35–7.50 (m, 9H, Ar–H), 7.77 (d, *J* = 8.2 Hz, 2H, Ar–H). ^13^C NMR (DMSO d6): δ 22.16, 42.49, 59.23, 69.79, 115.19, 122.13, 122.78, 124.46, 126.98, 127.96, 128.24, 128.37, 128.94, 131.44, 137.43, 154.49, 158.70, 160.51, 167.83. MS m/z (%): 449.93 (M^+^), 451.87 (M^+2^). Anal. Calcd for C_24_H_21_BrN_2_O_2_ (449.35): C, 64.15; H, 4.71; N, 6.23; %. Found: C, 64.16; H, 4.70; N, 6.23%.

##### *1-(3-(4-(Benzyloxy)phenyl)-5-(3-nitrophenyl)-4,5-dihydro-1H-pyrazol-1-yl)ethan-1-one* (9b).

Yield (55%), m.p. 127–129^◦^C. IR (KBr, cm^−1^): 1650 (C = O), 1610 (C = N). ^1^H NMR (DMSO d6, 400 MHz): δ 2.32 (s, 3H, CH_3_), 3.23 (dd, *J* = 18.0, 4.7 Hz, 1H, pyrazoline H_4A_), 3.88 (dd, *J* = 18.1, 12.0 Hz, 1H, pyrazoline H_4B_), 5.18 (s, 2H, CH_2_), 5.70 (dd, *J* = 12.0, 4.8 Hz, 1H, pyrazoline H_X_), 7.12 (d, *J* = 8.3 Hz, 2H, Ar–H), 7.33–7.50 (m, 5H, Ar–H), 7.63–7.66 (m, 2H, Ar–H), 7.74 (d, *J* = 8.3 Hz, 2H, Ar–H), 8.06 (s, 1H, Ar–H), 8.14 (d, *J* = 6.7 Hz, 1H, Ar–H). ^13^C NMR (DMSO d6): δ 22.12, 42.33, 59.21, 69.81, 115.57, 121.06, 122.71, 124.01, 128.21, 128.42, 128.95, 130.84, 132.85, 137.14, 144.98, 148.39, 154.57, 160.55, 167.99. HRMS (ESI): m/z calcd for C_24_H_21_N_3_O_4_: 416.16103 [M + H]^+^. Found 416.16154. Anal. Calcd for C_24_H_21_N_3_O_4_ (415.45): C, 69.39; H, 5.10; N, 10.11; %. Found: C, 69.38; H, 5.10; N, 10.12%.

##### *1-(3-(4-(Benzyloxy)phenyl)-5-(3,4-dimethoxyphenyl)-4,5-dihydro-1H-pyrazol-1-yl)ethan-1-one* (9c).

Yield (50%), m.p. 85–87 ^◦^C. IR (KBr, cm^−1^): 1655 (C = O), 1617 (C = N). ^1^H NMR (DMSO d6, 400 MHz): δ 2.30 (s, 3H, CH_3_CO), 3.11 (dd, *J* = 18.0, 4.2 Hz, 1H, pyrazoline H_4A_), 3.72 (s, 3H, OCH_3_), 3.73 (s, 3H, OCH_3_), 3.79 (dd, *J* = 18.1, 12.0 Hz, 1H, pyrazoline H_4B_), 5.18 (s, 2H, CH_2_), 5.47 (dd, *J* = 11.5, 4.2 Hz, 1H, pyrazoline H_X_), 6.65 (d, *J* = 8.0 Hz, 1H, Ar–H), 6.80 (s, 1H, Ar–H), 6.88 (d, *J* = 8.3 Hz, 1H, Ar–H), 7.10 (d, *J* = 8.5 Hz, 2H, Ar–H), 7.35–7.48 (m, 5H, Ar–H), 7.73 (d, *J* = 8.5 Hz, 2H, Ar–H). ^13^C NMR (DMSO d6): δ 22.22, 42.68, 56.06, 59.52, 69.82, 110.17, 115.57, 117.56, 124.38, 126.89, 128.19, 128.41, 128.76, 128.95, 135.50, 137.18, 148.41, 149.27, 154.42, 160.43, 167.66. MS m/z (%): 430.42 (M^+^). Anal. Calcd for C_26_H_26_N_2_O_4_ (430.50): C, 72.54; H, 6.09; N, 6.51; %. Found: C, 72.50; H, 6.10; N, 6.52%.

#### General procedure for synthesis of compounds 10a-c

A mixture of compounds (**8a-c)** (0.005 mol) was allowed to react with hydrazine hydrate (85%) (1 ml, 0.02 mol) for 6 h using ethanol as a solvent, then the mixture was concentrated in *vacuo*. After cooling, the obtained solid was filtered, dried and crystallized from ethanol.

##### 3-(4-(Benzyloxy)phenyl)-5-(3-bromophenyl)-4,5-dihydro-1H-pyrazole (10a)

Yield (56%), m.p. 86–88^◦^C. IR (KBr, cm^−1^): 3500 (NH). ^1^H NMR (DMSO d6, 400 MHz): δ 2.82 (dd, *J* = 16.3, 10.6 Hz, 1H, pyrazoline H_4A_ ), 3.44 (dd, *J* = 16.4, 10.7 Hz, 1H, pyrazoline H_4B_), 4.82 (dd, *J* = 10.6, 3.4 Hz, 1H, pyrazoline H_X_), 5.14 (s, 2H, CH_2_), 7.04 (d, *J* = 8.8 Hz, 2H, Ar–H), 7.30–7.36 (m, 2H, Ar–H), 7.39–7.42 (m, 3H, Ar–H), 7.46–7.49 (m, 4H, Ar–H), 7.56–7.58 (m, 3H, Ar–H and NH). ^13^C NMR (DMSO d6): δ 41.26, 63.12, 69.77, 115.38, 122.18, 126.99, 127.65, 128.17, 128.23, 128.35, 128.93, 128.99, 129.98, 130.49, 131.14, 131.43, 137.38, 159.16. MS m/z (%): 407.73 (M^+^), 409.39 (M^+2^). Anal. Calcd for C_22_H_19_BrN_2_O (407.31): C, 64.87; H, 4.70; N, 6.88; %. Found: C, 64.89; H, 4.73; N, 6.90%.

##### *3-(4-(Benzyloxy)phenyl*)-*5-(3-nitrophenyl)-4,5-dihydro-1H-pyrazole (10b)*

Yield (60%), m.p. 108–110^◦^C. IR (KBr, cm^−1^): 3515 (NH). ^1^H NMR (DMSO d6, 400 MHz): δ 2.87 (dd, *J* = 16.3, 10.6 Hz, 1H, pyrazoline H_4A_), 3.53 (dd, *J* = 16.4, 10.7 Hz, 1H, pyrazoline H_4B_), 5.00 (t, *J* = 9.4 Hz, 1H, pyrazoline H_X_), 5.14 (s, 2H, CH_2_), 7.04–7.06 (m, 2H, Ar–H), 7.34–7.46 (m, 5H, Ar–H), 7.58–7.70 (m, 4H, Ar–H), 7.87–7.88 (m, 1H, Ar–H), 8.15–8.16 (m, 1H, Ar–H), 8.26 (s, 1H, NH). ^13^C NMR (DMSO d6): δ 41.33, 63.03, 69.71, 115.36, 121.85, 122.53, 126.31, 127.49, 128.16, 128.33, 128.92, 130.48, 134.04, 137.40, 146.11, 148.30, 149.40, 159.02. MS m/z (%): 373.77 (M^+^). Anal. Calcd for C_22_H_19_N_3_O_3_ (373.41): C, 70.76; H, 5.13; N, 11.25; %. Found: C, 70.71; H, 5.16; N, 11.29%.

##### 3-(4-(Benzyloxy)phenyl)-5-(3,4-dimethoxyphenyl)-4,5-dihydro-1H-pyrazole (10c)

Yield (61%), m.p. 128–130^◦^C. IR (KBr, cm^−1^): 3514 (NH). ^1^H NMR (DMSO d6, 400 MHz): δ 2.81 (dd, *J* = 16.1, 11.3 Hz, 1H, pyrazoline H_4A_), 3.74 (s, 3H, OCH_3_), 3.75 (s, 3H, OCH_3_), 3.83 (dd, *J* = 16.1, 11.3 Hz, 1H, pyrazoline H_4B_), 4.75 (t, *J* = 10.8, 1H, pyrazoline H_X_), 5.14 (s, 2H, CH_2_), 6.91 (s, 2H, Ar–H), 7.01–7.05 (m, 3H, Ar–H), 7.33–7.36 (m, 1H, Ar–H), 7.41 (t, *J* = 7.4 Hz, 2H, Ar–H), 7.47 (d, *J* = 7.5 Hz, 2H, Ar–H), 7.57 (d, *J* = 8.3 Hz, 2H, Ar–H). ^13^C NMR (DMSO d6): δ 41.30, 55.90, 56.04, 63.95, 69.70, 110.92, 112.20, 115.32, 119.13, 126.71, 127.34, 128.16, 128.32, 128.92, 135.83, 137.43, 148.44, 149.16, 149.37, 158.85. HRMS (ESI): m/z calcd for C_24_H_24_N_2_O_3_: 389.18652 [M + H]^+^. Found 389.18654. Anal. Calcd for C_24_H_24_N_2_O_3_ (388.47): C, 74.21; H, 6.23; N, 7.21; %. Found: C, 74.20; H, 6.24; N, 7.20%.

### Biological screening

#### In vitro* anti-proliferative screening using MTT assay*

Cell viability was determined by a colorimetric assay using 3-(4,5-dimethylthiazol-2-yl)-2,5-diphenyltetrazolium bromide (MTT) (Sigma Co., St. Louis, USA) using four human tumor cell lines, namely; Colorectal carcinoma Colon cancer (HCT-116), mammary gland Breast cancer (MCF-7), hepatocellular carcinoma (HEPG-2) and the normal human lung fibroblast (WI38). Cell lines were obtained from ATCC via Holding company for biological products and vaccines (VACSERA), Cairo, Egypt. Doxorubicin (Dox.) is used as a standard anticancer drug. This colorimetric assay is based on the conversion of yellow (MTT) to a purple formazan derivative by mitochondrial succinate dehydrogenase in viable cells. This colorimetric assay was performed according to the reported method^36,37^.

#### In vitro* inhibition of epidermal growth factors receptor kinase (EGFR-TK) enzyme*

Enzyme inhibitory assays was carried out using the Kinase-Glo luminescent assay for the most active compounds **5a, 9b, 10a, 10b** and **10c** as described in the previous reports in the literature^53^.

#### Flow cytometric analysis of cell cycle distribution

Cell cycle analysis for compound **5a** was performed using the HepG-2 cell lines stained with propidium iodide (PI) and FACSCalibur flow cytometer as mentioned in previous reports^54^.

#### Analysis of cellular apoptosis

Apoptosis induction for **5a** was performed using the HepG-2 cell lines and well-established Annexin 5-FITC/PI detection kit similar to the reported procedures^55^.

#### Gene expression analysis (RNA extraction and real-time RT-PCR for tested genes)

Gene expression analysis was performed by using (iScriptTM OneStep RT-PCR Kit with SYBR® Green) from Bio-Rad according to reported procedure^56^.

#### Intracellular ROS accumulation assay

Intracellular reactive oxygen species (ROS) were detected by the EIAab ROS ELISA kit according to the reported method^57^.

#### Molecular modeling methodology

Enzyme inhibition assay revealed compounds **5a, 10b** as the most active derivatives while compound **5a** demonstrated much higher activity in comparison to the reference compound gefitinib. In order to investigate the binding mode of these compounds molecular docking was utilized to obtain insights on their interaction with the active site of EGFR. Hence, its crystal structure was obtained from PDB using the code: 4WQK. The retrieved 3D structures were prepared using Protein Repair and Analysis Server, where bond orders were assigned, missing atoms were added, hydrogen bonds were optimized, charges were corrected^58^. Then, water molecules and co-crystallized ligand were removed. The prepared PDB file of the protein was loaded in protein preparation module integrated in PyRX software for virtual screening^59^, where it was converted to Pdbqt files and the active site was defined according as grid box size around the cocrystallized ligand with box size 26 × 26 × 26 and the coordinates were X: 0, Y: 197, and Z: 21.

Compounds (**5a, 10a, 10b** and **gefitinib**) were sketched using Marvin sketch version 21.17.0, ChemAxon (https://www.chemaxon.com) and saved as mol files which were loaded to the ligand preparation module integrated in PyRX and converted to Pdbqt. The molecular docking was done using Autodock vina as the docking engine, where exhaustiveness was set as 12 and the number of poses was three. The software ranked the poses according to their binding affinity (kcal/mol), and the docked poses were analyzed to determine their interaction profiles with amino acid residues in the binding site using LigPlot + , which produces 2D presentation of ligand protein complex^60^.

### Supplementary Information

Supplementary Information.

## Data Availability

All data generated or analyzed during this study are included in this published article and its supplementary information file.
