# Supplementary material for: New oxadiazole and pyrazoline derivatives as anti-proliferative agents targeting EGFR-TK: design, synthesis, biological evaluation and molecular docking study
Source: Sci Rep. 2024 Mar 5;14:5474. doi: 10.1038/s41598-024-55046-0 (PMC10915170; doi:10.1038/s41598-024-55046-0)
Supplement: Supplementary file 1 — Supplementary Information. [file 41598_2024_55046_MOESM1_ESM.docx]

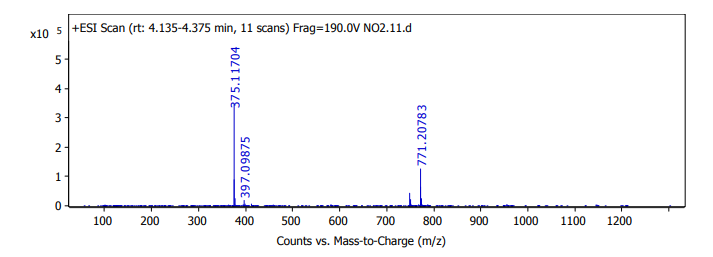


Chart 1: high resolution mass (HRMS) of compound **5a (**C_22_H_18_N_2_O_2_S)


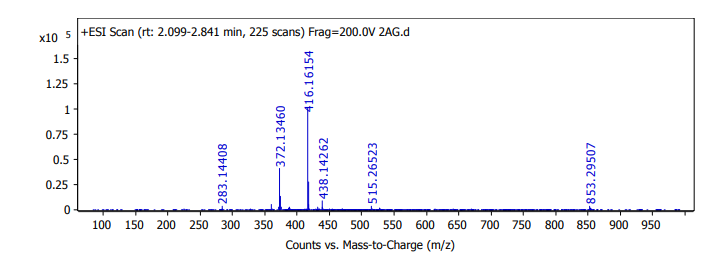


Chart 2: high resolution mass (HRMS) of compound **9b (**C_24_H_21_N_3_O_4_)


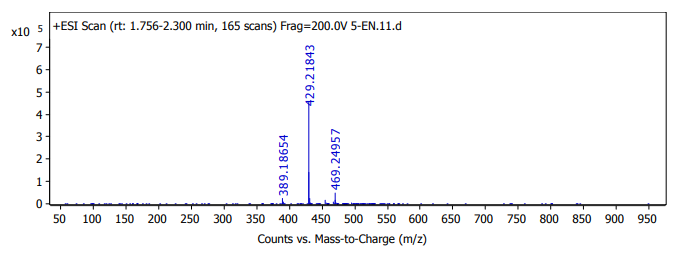


Chart 3: high resolution mass (HRMS) of compound **10c (**C_24_H_24_N_2_O_3_)


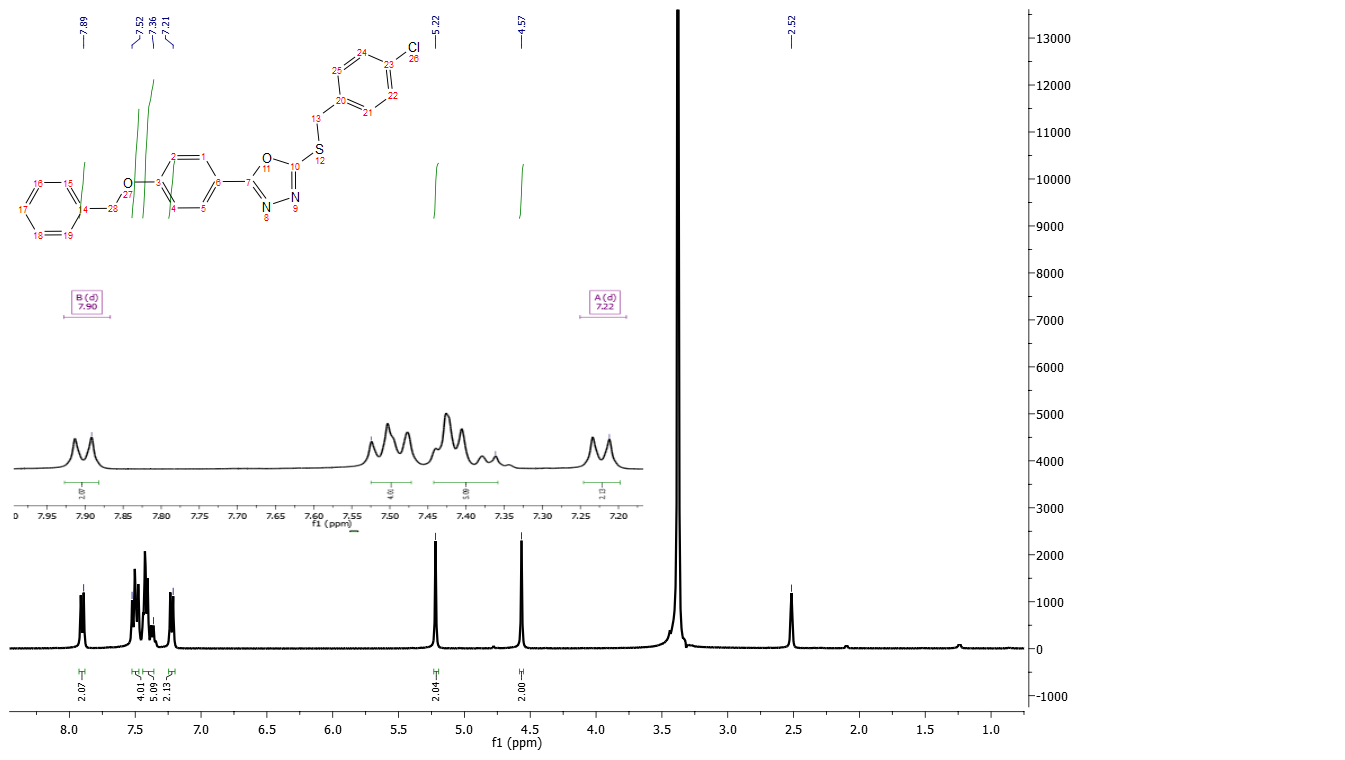


Chart 4: ^1^H NMR (DMSO d6, 400 MHz) of compound **5b**


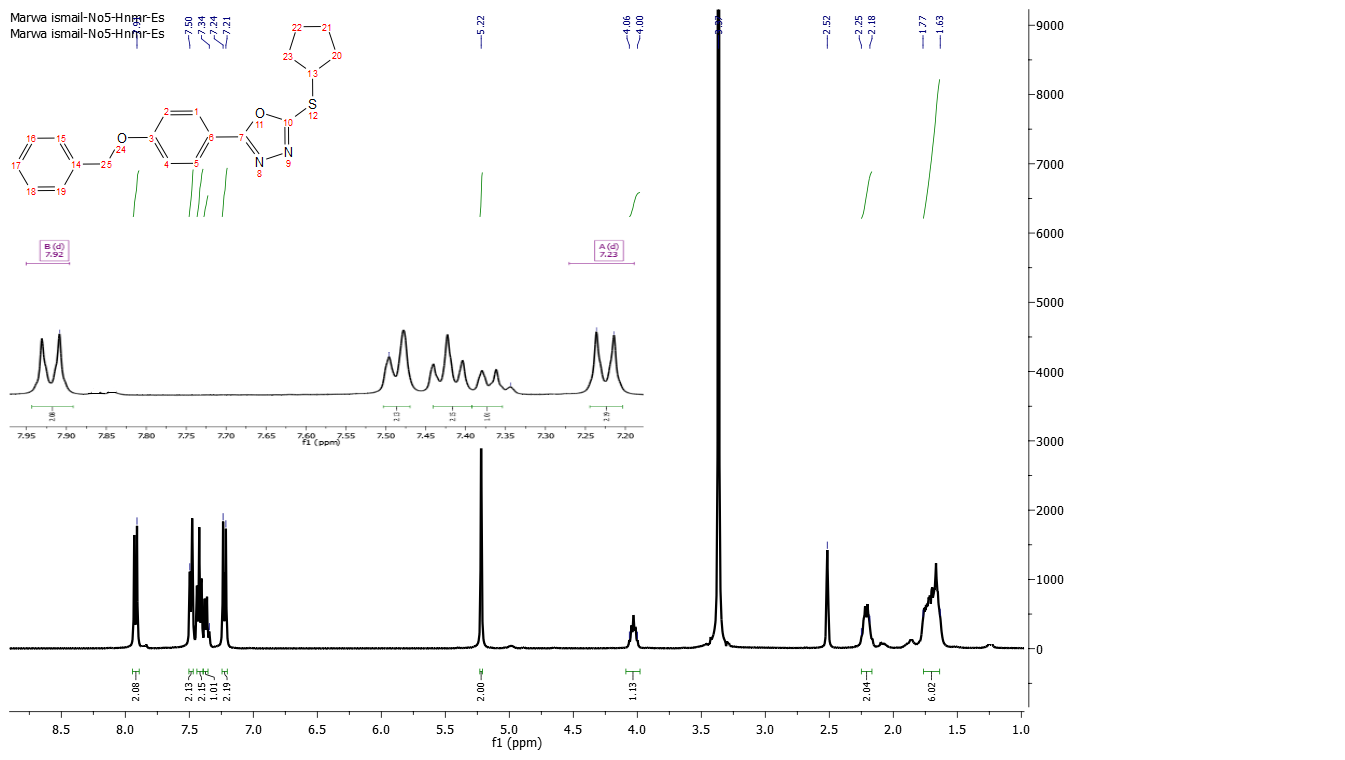
Chart 5: ^1^H NMR (DMSO d6, 400 MHz) of compound **5e**

Chart 6: ^1^H NMR (DMSO d6, 400 MHz) of compound **5f**

Chart 7: ^13^C NMR (DMSO d6) of compound **5a**

Chart 8: ^13^C NMR (DMSO d6) of compound **5b**

Chart 9: ^13^C NMR (DMSO d6) of compound **5f**


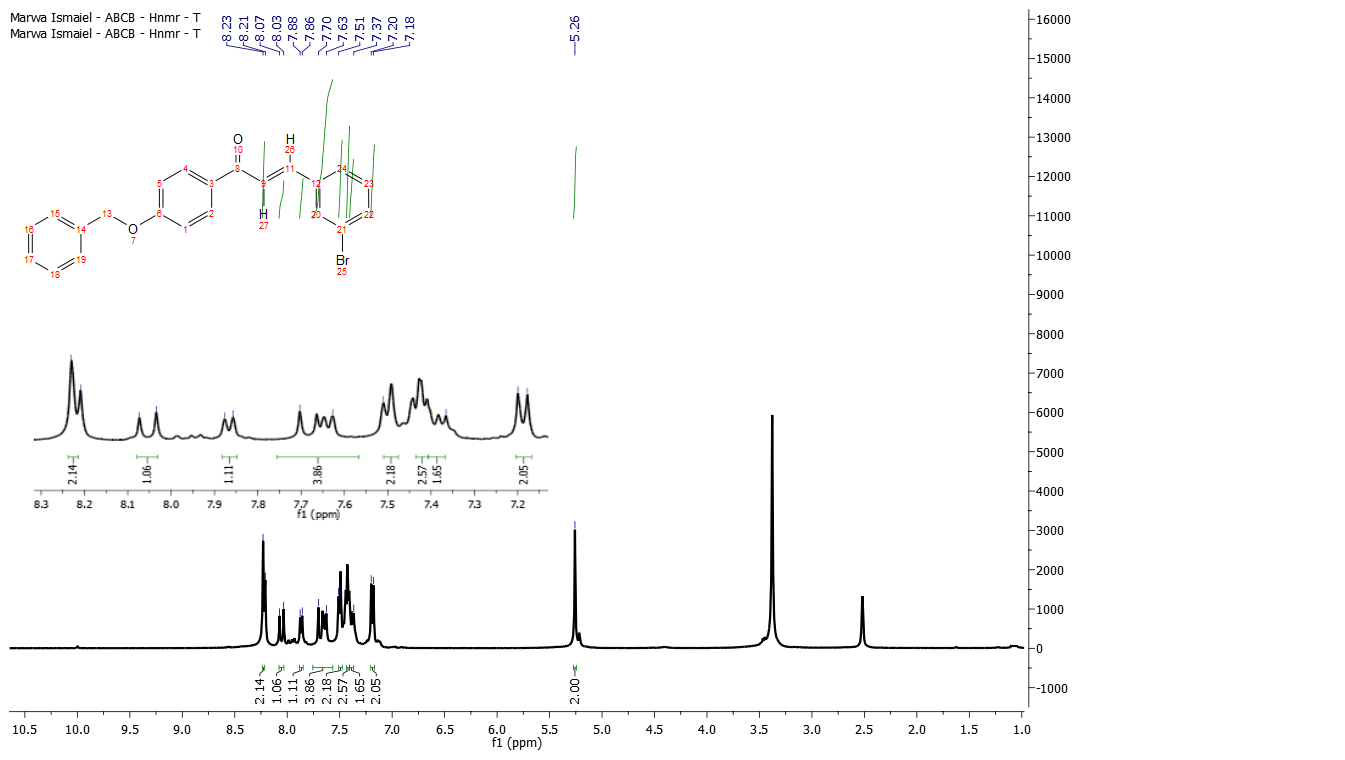
Chart 10: ^1^H NMR (DMSO d6, 400 MHz) of compound **8a**


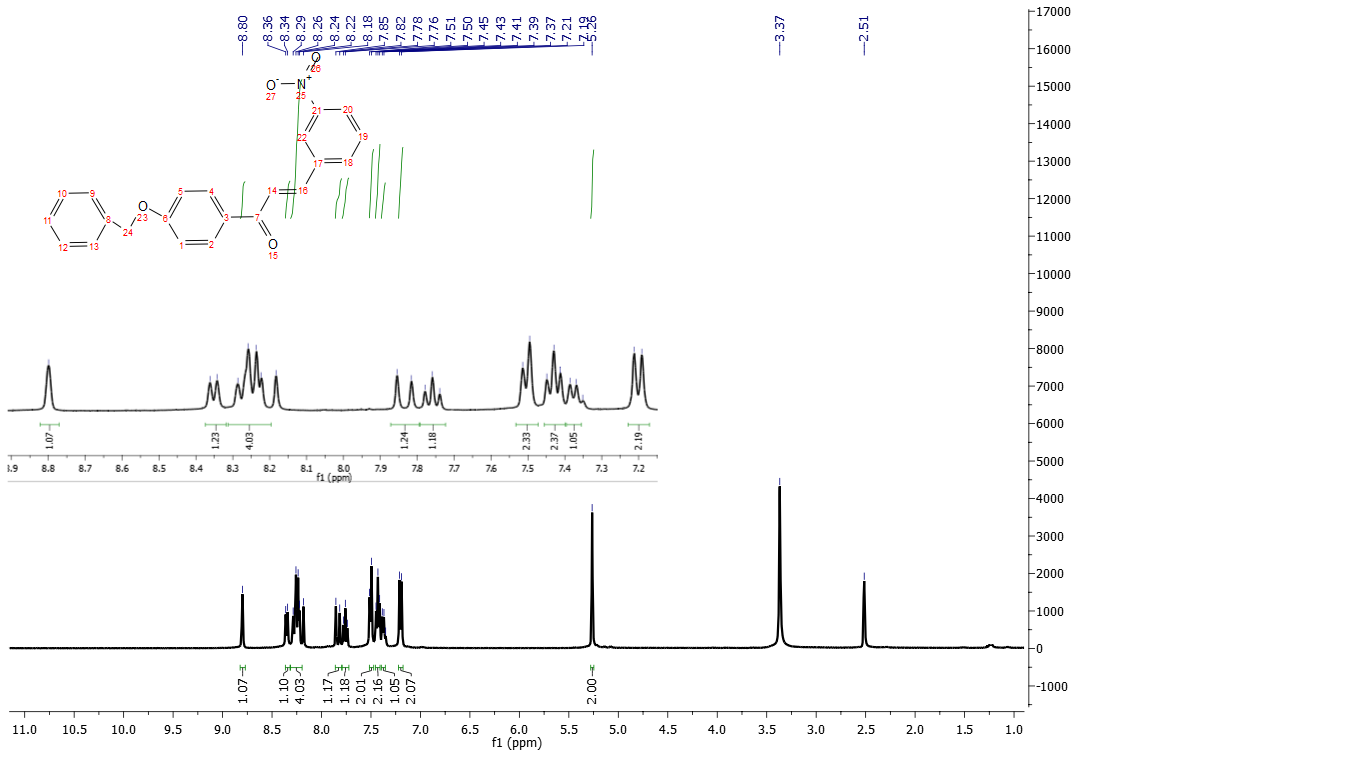


Chart 11: ^1^H NMR (DMSO d6, 400 MHz) of compound **8b**

Chart 12: ^1^H NMR (DMSO d6, 400 MHz) of compound **8c**


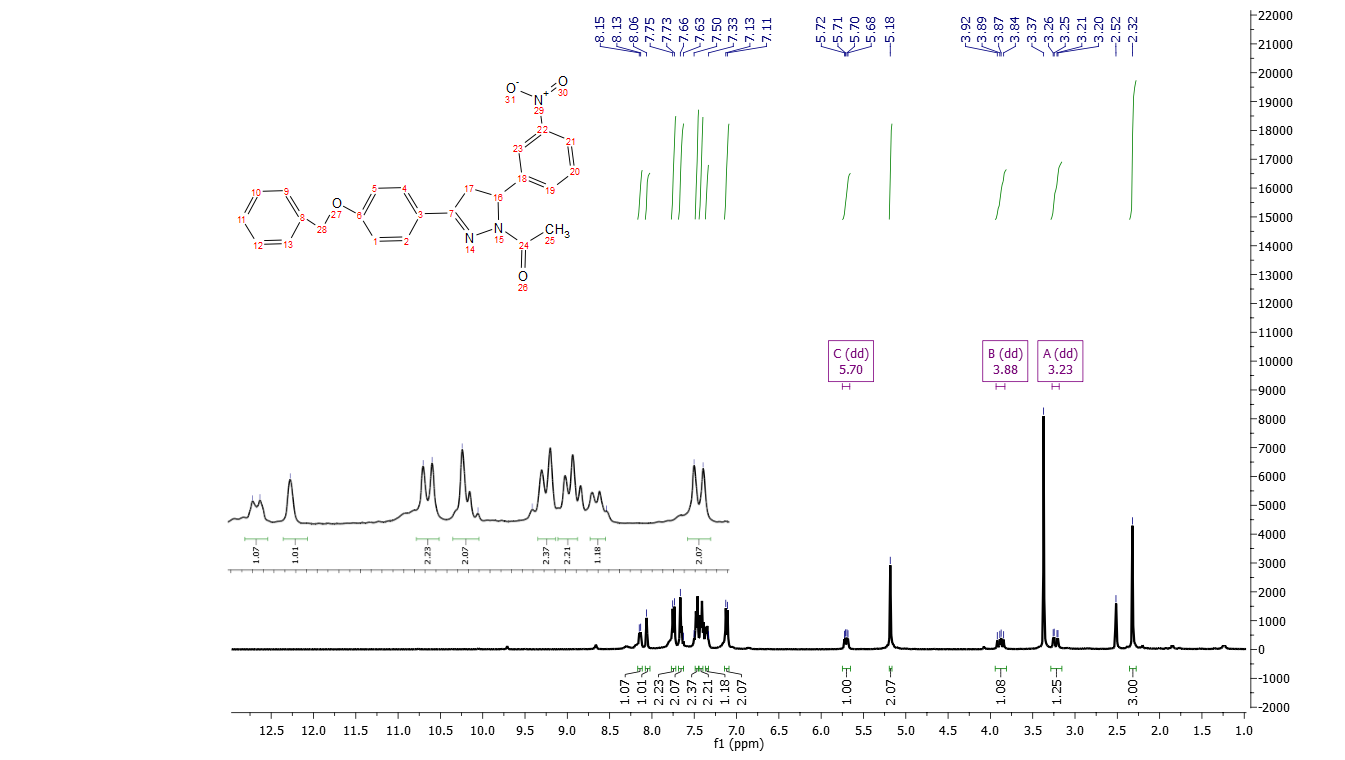


Chart 13: ^1^H NMR (DMSO d6, 400 MHz) of compound **9b**


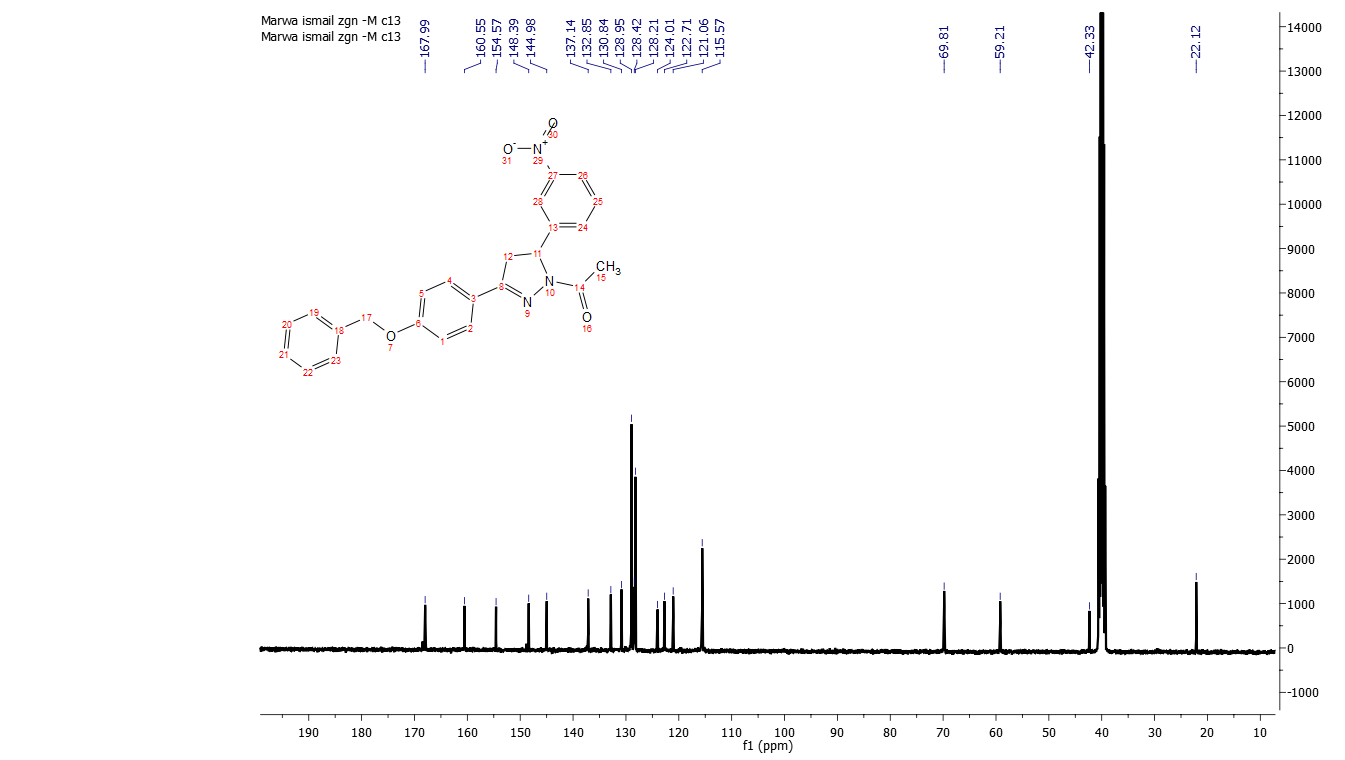


Chart 14: ^13^C NMR (DMSO d6) of compound **9b**


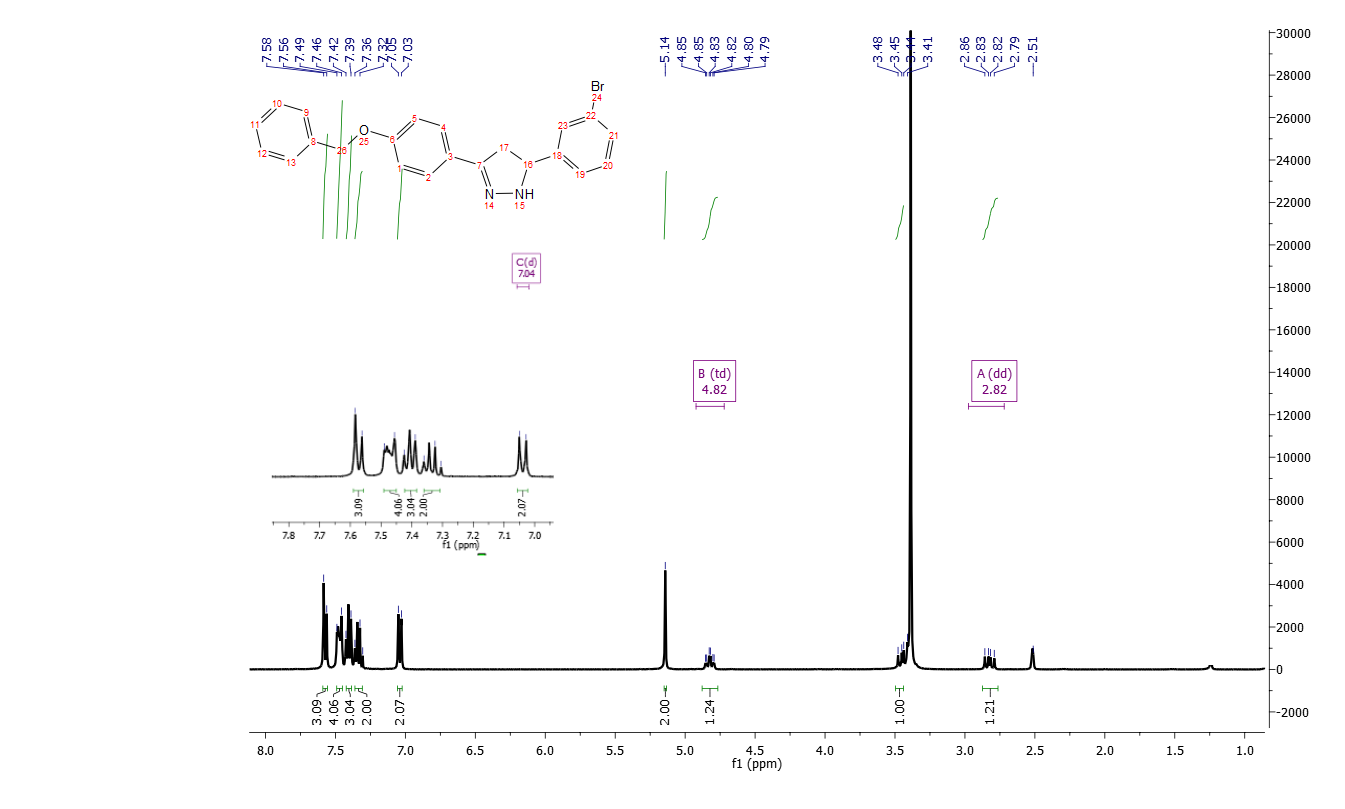
Chart 15: ^1^H NMR (DMSO d6, 400 MHz) of compound **10a**

Chart 16: ^13^C NMR (DMSO d6) of compound **10b**

Chart 17: ^1^H NMR (DMSO d6, 400 MHz) of compound **10c**
